# The endosteal niche regulates breast cancer cell dormancy in bone: identification of new molecular determinants

**DOI:** 10.1038/s41413-026-00535-3

**Published:** 2026-05-14

**Authors:** Antonio Maurizi, Maria Salbini, Michela Ciocca, Marzia Rea, Giuseppe D. Tocchini-Valentini, Matilde Merolle, Hanna Taipaleenmäki, Christina Møller Andreasen, Manuela Pellegrini, Anna Teti

**Affiliations:** 1 Department of Biotechnological and Applied Clinical Sciences, University of L’Aquila, L’Aquila, Italy; 2 Institute of Biochemistry and Cell Biology, National Council of Research, Monterotondo (Rome), Italy; 3 European Mouse Mutant Archive (EMMA), INFRAFRONTIER-IMPC, Mouse Clinic, National Council of Research, Monterotondo (Rome), Italy; 4 Institute of Musculoskeletal Medicine, LMU University Hospital, LMU Munich, Planegg-Martinsried, Germany; 5 Musculoskeletal University Center Munich, LMU University Hospital, LMU Munich, Planegg-Martinsried, Germany; 6 Research Unit of Pathology, Department of Clinical Research, University of Southern Denmark, Odense, Denmark

**Keywords:** Bone cancer, Cancer

## Abstract

Cellular dormancy compromises the long-term survival of breast cancer patients. Bone represents a frequent site for metastasis, where Spindle-shaped N-cadherin^+^CD45^−^ osteoblasts (SNOs) hold dormant metastatic cells in the endosteal niche with a Notch2-dependent mechanism. In this work, we excluded the involvement of Notch1 in SNO-induced breast cancer cellular dormancy by immunofluorescence/immunohistochemistry and molecular approaches, using breast cancer tissues and cell lines. RNAdSeq in human bone metastatic MDA-MB231 breast cancer cells sorted for Notch1^HIGH^ and Notch2^HIGH^ expression demonstrated that, compared to their low counterpart, only Notch2^HIGH^ cells expressed enriched pathways relevant for the metastatic process, including pluripotency and Hematopoietic Stem Cell (HSC) gene signatures. They expressed the HSC-associated genes *CXCR4*, *CD34* and *TIE2* and MDA-MB231 cells enriched in the encoded proteins showed lesser proliferation ability. Reduced incidence of osteolytic lesions was induced by CXCR4^HIGH^ cells intratibially injected in immunocompromised mice, while lower lesion extension was induced by CXCR4^HIGH^ and TIE2^HIGH^ injected cells compared to CXCR4^LOW^ and TIE2^LOW^ cells. Notch2^HIGH^ cells were enriched in endoplasmic reticulum stress and unfolded protein response genes and overexpressed the CD177 protein, while the CD177 ligands, Plaur, Itgam and Ceacam 1, were highly expressed in SNOs. Kaplan-Meier plots showed positive correlation between high expression of CD177, ITGAM and CEACAM 1 - but not PLAUR - and overall survival of patients. CD177^HIGH^ cells were also CXCR4^HIGH^, CD34^HIGH^ and Notch2^HIGH^ and proliferated less than CD177^LOW^ cells. These results support the relevance of Notch2 in SNO-mediated cellular dormancy and identified new pathways implicated in bone metastatic breast cancer cell quiescence.

## Introduction

Cellular dormancy is the mechanism whereby single metastatic cancer cells lodge in the host tissue remaining cell cycle arrested for long time.^1^ These cells have high risk of reactivation, relapsing overt metastases also after decades from apparent cancer healing.^2^ The bone marrow is a preferential site of breast cancer (BrCa) metastases,^3^ and cellular dormancy is a typical event that prevents permanent cancer remission after therapy.^4^ Understanding the cellular and molecular mechanisms of dormancy in the bone marrow could provide a solid background to identify ways to permanently eradicate dormant cells or to prevent their long-term reactivation and spreading in bone and other organs, lastingly curing BrCa.

The endosteum is the layer of cells that covers the inner surface of the bone facing the bone marrow.^5^ It is mainly made by osteogenic cells, called osteoblasts when they are active in the bone formation process, and lining cells when they become quiescent at the end of bone deposition.^6^ They also include a subset of cells supporting long-term haematopoiesis and regulating the size of the myeloid cell pool.^7^ These cells are known as Spindle-shaped N-cadherin^+^CD45^−^ osteoblasts (SNOs) and represent a Haematopoietic Stem Cell (HSC) niche.^8^ An increase of SNOs is associated with a parallel increase of HSCs. Furthermore, the most primitive Long-Term (LT)-HSCs bind SNOs with the adherens junction molecules, N-cadherin and β-catenin, observed at the SNO-LT-HSC interface.^8^ The SNO-LT-HSC interaction is regulated by bone morphogenetic proteins^8^ and Parathyroid Hormone (PTH)^9^ signals. Notably, PTH-activated osteoblasts express high levels of the Notch ligand, Jagged1, and support the HSC pool expansion with a mechanism involving Notch1 expressed by HSCs.^10^

Like LT-HSCs, dormant BrCa cells interact with SNOs at the endosteal interface.^11^ In fact, we previously demonstrated that single, non-proliferating BrCa cells lodge in proximity of the endosteal surface, remaining quiescent for long time. They also compete with HSCs for bone marrow engraftment, showing molecular similarities with this stem cell pool.^11^ SNOs are enriched in the endosteal areas where dormant BrCa cells are recruited, show lower expression of osteoblast-specific genes, and are confirmed to be enriched in the Notch ligand, Jagged 1.^11,12^

Among the Notches expressed by BrCa cells, Notch2 promotes their interactions with SNOs, with a mechanism blunted by the γ-secretase inhibitor, dibenzazepine.^11^ Notch2^HIGH^ BrCa cells represent a small population within the tumour, exhibiting also stem-like features,^13^ and are observed likewise in human BrCa tissues in which they show the ability to lodge as single cells at the endosteal surface of human bone metastases.^14^ In our previous study, we observed that Notch1 is also more expressed in a subset of BrCa dormant cells, although at a lower level compared to Notch2, but its contribution to the interaction with SNOs was not clarified. In contrast, Notch3 and Notch4 appeared irrelevant.^11^

Notch1 and Notch2 are very similar paralog receptors that signal via the conserved canonical Notch pathway. Despite their shared signalling mechanism, they often exhibit distinct, non-redundant, or complementary functional roles across specific developmental processes and pathological conditions.^15^ In this study, we aimed at structuring the molecular features of BrCa cells lodging the bone environment, focusing on the Notch1 and Notch2 pathways as determinants of the dormant phenotype. To this end, we characterized the Notch1- and Notch2-related molecular phenotypes of various BrCa cell lines, selecting the MDA-MB231 human cell line as leading cellular tool for the study. We performed a global transcriptomic analysis to examine the Gene Ontology (GO) and the Kyoto Encyclopedia of Genes and Genomes (KEGG) pathways and establish the Notch1^HIGH^ and Notch2^HIGH^ dormant BrCa cell molecular signatures. From this analysis, we extrapolated that Notch2^HIGH^ cells express a dominant signature determining the molecular alterations of dormant cells and preserving their pluripotent phenotype and HSC-like gene expression. Furthermore, we identified the molecular interactome binding Notch2^HIGH^ BrCa cells to SNOs and, through functional studies, we recognized the glycosylphosphatidylinositol (GPI)-linked surface glycoprotein, CD177, and CXCR4 as potential molecular pathways involved in the BrCa dormant behaviour in the bone microenvironment.

## Results

### Notch1 and Notch2 expression in BrCa cells and their role in the interaction with SNOs

**Fig. 1:**
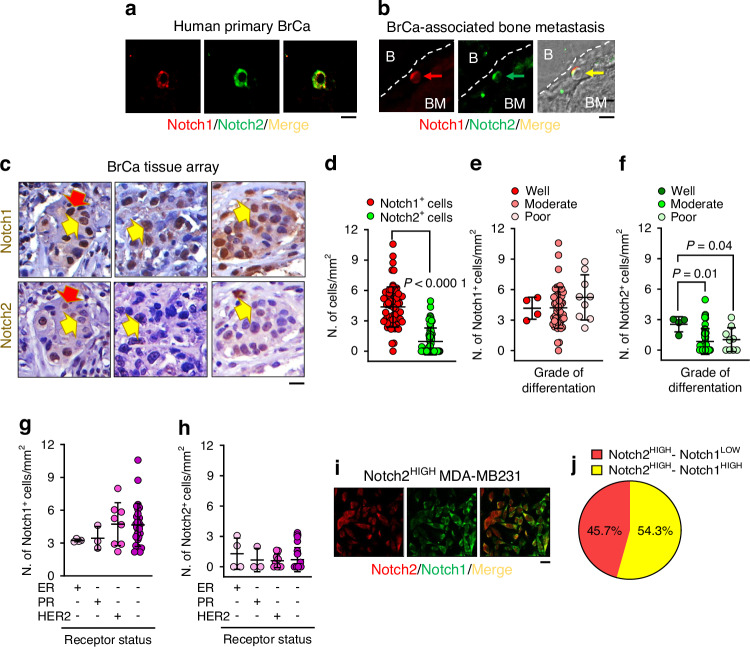
Expression of Notch1 and Notch2 in BrCa. **a** Immunofluorescence detection of a Notch1^+^/Notch2^+^ cell in a primary BrCa sample. **b** Immunofluorescence detection of a Notch1^+^/Notch2^+^ cell (arrow) located near the endosteal surface (dotted line) in an associated bone metastasis. B: bone; BM: bone marrow. **c** Immunohistochemical detection of Notch1^+^ and Notch2^+^ cells in a BrCa tissue array. Yellow arrows: Notch1^+^/Notch2^−^ cells; red arrows: Notch1^+^/Notch2^+^ cells. **d** Quantification of Notch1^+^ and Notch2^+^ cells in the tissue array analysed in (**c**). **e** Stratification of Notch1^+^ and **f** Notch2^+^ cells according to the grade of BrCa differentiation. **g** Distribution of Notch1^+^ and **h** Notch2^+^ cells in BrCa tissue array samples according to the oestrogen receptor (ER), progesterone receptor (PR) and human epidermal growth factor receptor 2 (HER2) status. **i** Co-expression of Notch1 in MDA-MB231 BrCa cells sorted for high expression of Notch2 (Notch2^HIGH^). **j** Evaluation of the percentage of MACS-sorted Notch2^HIGH^ MDA-MB231 cells expressing low (red) or high (yellow) levels of Notch1. Results are representative (**a**–**c**, **i**) (bars = 10 µm) or the mean ± SD (**d**–**h**, **j**) of 3 independent samples per group. Statistics: unpaired *t* test

To investigate whether Notch1 and Notch2 were expressed in BrCa cells, we performed double immunofluorescence for Notch1 and Notch2 in human primary BrCa tissue and associated bone metastasis, derived from our internal tissue biobank. Figure 1a and Fig. S1a show primary cancer cell co-expressing Notch1 and Notch2. Similar Notch1 and Notch2 co-staining was observed in single cancer cells located near the endosteum in a sample of human BrCa-associated bone metastasis (Fig. 1b, Fig. S1b). Furthermore, single immunohistochemistry for Notch1 and Notch2 in serial sections of human BrCa tissue array (Table S1) revealed a higher number of Notch1^+^ cells compared to Notch2^+^ cells in contiguous sections (Fig. S1c). Most Notch1^+^ cells were Notch2^-^ (yellow arrows), while only a small number of them was positive to both Notches (red arrows) (Fig. 1c). Quantitative evaluations confirmed that Notch1^+^ cells were more numerous in primary BrCa compared to Notch2^+^ cells (Fig. 1d), with no differences associated with the grade of tumour differentiation (Fig. 1e). In contrast, Notch2^+^ cells were less numerous in moderately and poorly differentiated cancers vs well differentiated cancers (Fig. 1f).

Notch1 and Notch2 expression showed no association with the receptor status of the human primary cancers (Fig. 1g, h). Furthermore, in vitro analysis on Notch2^+^ MDA-MB231 cells demonstrated that 54.3% of them were also Notch1^+^ (Fig. 1i, j), prompting us to investigate the roles of these two molecular determinants in cellular dormancy.

**Fig. 2:**
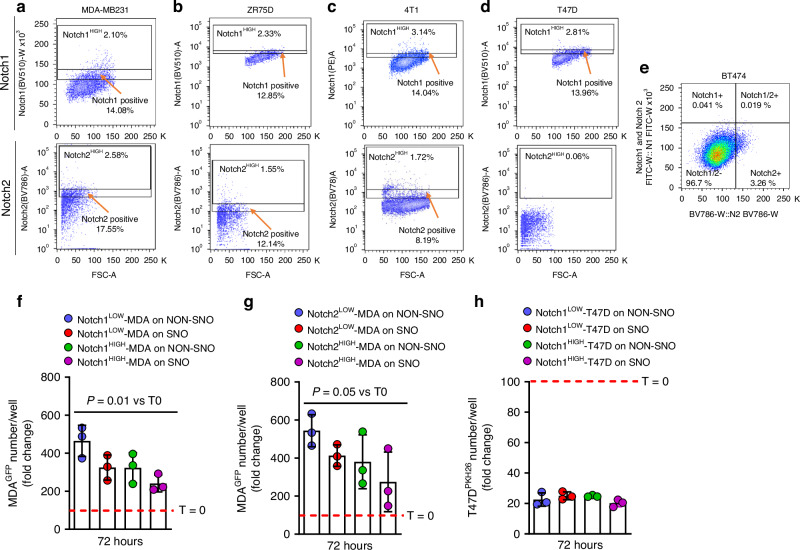
Expression of Notch1 and Notch2 in breast cancer cells and interaction with SNOs. **a** Human MDA-MB231, **b** human ZR75D, **c** mouse 4T1, **d** human T47D and **e** human BT747 BrCa cell lines were analysed by flow cytometry for their expression of Notch1 and Notch2. **f** Quantification of human MDA-MB231 GFP^+^ BrCa cells (MDA^GFP^) MACS-sorted for low or high expression of Notch1 after 72 h of culture on SNO and NON-SNO monolayers. **g** Quantification of human MDA-MB231 GFP^+^ BrCa cells (MDA^GFP^) MACS-sorted for low or high expression of Notch2 after 72 h of culture on SNO and NON-SNO monolayers. **h** Quantification of human T47D BrCa cells labelled with the PKH26 membrane dye, MACS-sorted for low or high expression of Notch1 after 72 h of culture on SNO and NON-SNO monolayers. Results are (**a**–**e**) representative or (**f**–**h**) the mean ± SD of 3 independent experiments. Statistics: unpaired *t*-test

Our previous reports^11,12^ demonstrated that human MDA-MB231 and mouse 4T1 cell lines include small populations of Notch2^HIGH^ cells. Here we extended the analysis, and flow cytometry showed that a small population of the total human MDA-MB231 were Notch1^HIGH^ and Notch2^HIGH^ (Table S2, Fig. 2a). Similar Notch2^HIGH^/Notch1^HIGH^ coexistence was observed in small populations of other BrCa cell lines, including human ZR75D cells (Table S2, Fig. 2b) and mouse 4T1 cells (Table S2, Fig. 2c). In contrast, human T47D cells were Notch2^-^ and presented a small Notch1^HiGH^ population (Table S2, Fig. 2d), while human BT474 cells were Notch1^-^ with a small Notch2^HIGH^ population (Table S2, Fig. 2e) and human MCF-7 cells were negative to both Notches (Table S2). These observations confirmed the paucity of the Notch1^HIGH^ and Notch2^HIGH^ populations in several BrCa cell lines and demonstrated heterogeneity in their expression.

We have previously demonstrated that Notch2^HIGH^ MDA-MB231 cells proliferate less than Notch2^LOW^ cells when interacting with SNOs.^11^ To investigate if Notch1 played a similar role as Notch2, we plated MDA-MB231 GFP^+^ cells MACS-sorted for Notch1^HIGH^, Notch1^LOW^, Notch2^HIGH^ and Notch2^LOW^ onto NON-SNO and SNO monolayers, and performed GFP^+^ cell counting after 72 h of co-culture. Results demonstrated that Notch1^HIGH^ and Notch2^HIGH^ MDA-MB231 cells were less numerous compared to Notch1^LOW^ and Notch2^LOW^ cells plated on SNOs and on NON-SNOs, and to Notch1^HIGH^ and Notch2^HIGH^ cells plated on NON-SNOs (Fig. 2f, g). However, given that about 50% of the two sorted populations shared high levels of both Notch1 and Notch2 (Fig. 2a), this experiment did not clarify the roles of each of the two Notches in the SNO-induced MDA-MB231 cell dormancy. Therefore, to remove the confounding effects of the co-expression of Notch1 and Notch2, we performed a similar experiment using T47D cells sorted for Notch1^HIGH^, which were shown to be negative for Notch2 (Fig. 2d). In this context, cells were loaded with the impermeant cell surface fluorescent dye PKH26, whose fluorescence decreases at each doubling of the cells. Results showed a similar proliferation rate of PKH26-positive cells in each condition tested, demonstrated by the reduced number of PKH26-positive cells (Fig. 2i), mitigating a potential role of Nocth1 in SNO-induced cancer cell dormancy.

### RNAdSeq and Gene Ontology (GO) analysis of the differentially expressed mRNAs in Notch1 and Notch2 HIGH and LOW cells

To further characterize the molecular and functional differences between the Notch1^HIGH^ and Notch2^HIGH^ cell subsets, we settled a broad approach by RNAdSeq analysis, focusing on the MDA-MB231 as cellular model. We used a “systemic and systematic” strategy to identify transcriptional differences between MDA-MB231 cells MACS-sorted for Notch1^HIGH^, Notch1^LOW^, Notch2^HIGH^ and Notch2^LOW^ expression. We found 522 genes differentially expressed in the Notch1^HIGH^ vs Notch1^LOW^ cells and 1799 genes differentially expressed in the Notch2^HIGH^ vs Notch2^LOW^ cells (Fig. S2a, b). Then, by bioinformatics analysis the upregulated and downregulated transcripts were normalized and grouped according to the represented biological processes (BPs), molecular functions (MFs) and cellular components (CCs) GO terms and pathways, focusing on the differentially expressed transcripts identified for each condition tested (Supplementary Data file 1, 2). The total GO and KEGG pathway analyses are reported in the Supplementary Data files 3–6.

**Fig. 3:**
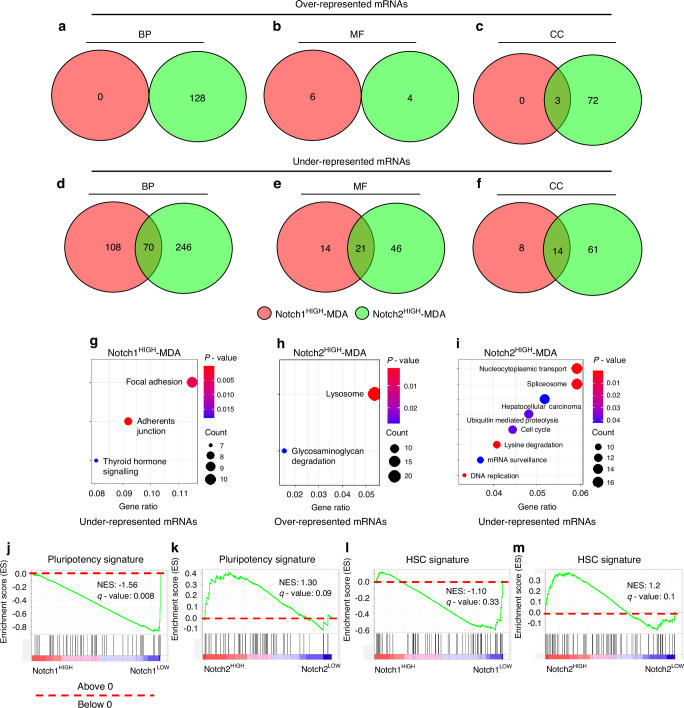
Bioinformatics analysis of RNAdSeq data. MACS-sorted Notch1^HIGH^ and Notch2^HIGH^ MDA-MB231 cells were subjected to RNAdSeq and analysed for differential mRNA expression. **a** Schematic representation of BP, **b** MF and **c** CC over-represented GO terms and **d** BP, **e** MF and **f** CC under-represented GO terms. **g** KEGG pathway analysis of under-represented mRNAs in Notch1^HIGH^ cells. **h** KEGG pathway analysis of over-represented mRNAs in Notch2^HIGH^ cells. **i** KEGG pathway analysis of under-represented mRNAs in Notch2^HIGH^ cells. **j** Pluripotency gene signature in Notch1^HIGH^ and Notch1^LOW^ cells and **k** in Notch2^HIGH^ and Notch2^LOW^ cells. **l** HSC gene signature in Notch1^HIGH^ and Notch1^LOW^ cells and **m** in Notch2^HIGH^ and Notch2^LOW^ cells. Results are representative of 3 independent cell preparations per group evaluated by the Benjamini-Hochberg adjustment *P*-value procedure

As first step of comparison, we identified statistically significant GO terms associated with upregulated and downregulated transcripts found in Notch1^HIGH^ and Notch2^HIGH^ cells vs their LOW counterparts (Tables S3 and S4), then we searched for shared GO terms between Notch1^HIGH^ and Notch2^HIGH^ cell populations. Interestingly, no shared upregulated biological processes and molecular functions, and only 3 shared cellular components GO terms, including *collagen-containing extracellular matrix*, *basement membrane* and *endoplasmic reticulum lumen* were observed between the two groups (Fig. 3a–c, Table 1).

**Table 1:** Shared upregulated GO terms in the Notch1^HIGH^ and Notch2^HIGH^ MDA-MB231 cells

ID	Description	*P*.adjust
Shared Cellular Components (CCs)		
GO:0062023	collagen-containing extracellular matrix	0.030 611 25
GO:0005604	basement membrane	0.030 611 25
GO:0005788	endoplasmic reticulum lumen	0.030 611 25

In contrast, many more GO terms associated with downregulated transcripts were shared between Notch1^HIGH^ and Notch2^HIGH^ cells, with 70 shared BP terms, 21 MF terms and 14 CC terms (Fig. 3d–f). Among these shared terms, the majority were associated with DNA replication, transcription, modification, organization and binding, suggesting a negative impact on cell cycle and proliferation (Table 2).

**Table 2:** Shared dowregulated GO terms in the Notch1^HIGH^ and Notch2^HIGH^ MDA-MB231 cells

ID	Description	*P*.adjust
Shared Processes (BPs)		
GO:0016569	covalent chromatin modification	5.276 3E-14
GO:0016570	histone modification	5.649 1E-13
GO:0018205	peptidyl-lysine modification	1.060 2E-12
GO:0045787	positive regulation of cell cycle	7.143 6E-07
GO:0006338	chromatin remodeling	1.099 1E-06
GO:0016571	histone methylation	1.840 5E-06
GO:0090068	positive regulation of cell cycle process	2.037 6E-06
GO:0018393	internal peptidyl-lysine acetylation	8.367 5E-06
GO:0051568	histone H3-K4 methylation	8.506 9E-06
GO:0006475	internal protein amino acid acetylation	8.893E-06
GO:0018394	peptidyl-lysine acetylation	1.342 2E-05
GO:0016573	histone acetylation	1.427 6E-05
GO:0006479	protein methylation	3.769 4E-05
GO:0008213	protein alkylation	3.769 4E-05
GO:0071824	protein-DNA complex subunit organization	4.618 6E-05
GO:0006473	protein acetylation	4.810 2E-05
GO:0006333	chromatin assembly or disassembly	5.363 2E-05
GO:0006352	DNA-templated transcription, initiation	0.000 133 08
GO:0030518	intracellular steroid hormone receptor signaling pathway	0.000 157 96
GO:0016925	protein sumoylation	0.000 257 23
GO:0080182	histone H3-K4 trimethylation	0.000 260 08
GO:0043543	protein acylation	0.000 292 58
GO:0043401	steroid hormone mediated signaling pathway	0.000 317 73
GO:0030219	megakaryocyte differentiation	0.000 424 79
GO:0033143	regulation of intracellular steroid hormone receptor signaling pathway	0.000 451 49
GO:0019080	viral gene expression	0.000 628 46
GO:0043967	histone H4 acetylation	0.001 030 46
GO:0006323	DNA packaging	0.001 181 98
GO:1904837	beta-catenin-TCF complex assembly	0.002 064 77
GO:0034728	nucleosome organization	0.002 186 26
GO:0009755	hormone-mediated signaling pathway	0.002 186 26
GO:0001654	eye development	0.002 240 09
GO:0018023	peptidyl-lysine trimethylation	0.002 418 95
GO:0150063	visual system development	0.002 521 12
GO:0071383	cellular response to steroid hormone stimulus	0.002 952 06
GO:0006367	transcription initiation from RNA polymerase II promoter	0.002 957 52
GO:0060968	regulation of gene silencing	0.002 976 27
GO:0048880	sensory system development	0.003 080 88
GO:0006476	protein deacetylation	0.003 544 92
GO:0006096	glycolytic process	0.004 550 45
GO:0006757	ATP generation from ADP	0.004 862 23
GO:0060964	regulation of gene silencing by miRNA	0.004 862 23
GO:0034329	cell junction assembly	0.006 056 56
GO:0060147	regulation of posttranscriptional gene silencing	0.006 252 5
GO:0060966	regulation of gene silencing by RNA	0.006 252 5
GO:0035601	protein deacylation	0.007 295 35
GO:0030522	intracellular receptor signaling pathway	0.007 910 2
GO:0046031	ADP metabolic process	0.007 913 75
GO:1900034	regulation of cellular response to heat	0.008 425 4
GO:0031497	chromatin assembly	0.008 425 4
GO:0098732	macromolecule deacylation	0.009 295 29
GO:0030521	androgen receptor signaling pathway	0.012 206 99
GO:0006165	nucleoside diphosphate phosphorylation	0.013 016 92
GO:0046939	nucleotide phosphorylation	0.014 424 01
GO:0042771	intrinsic apoptotic signaling pathway in response to DNA damage by p53 class mediator	0.014 825 18
GO:0009135	purine nucleoside diphosphate metabolic process	0.015 014 34
GO:0009179	purine ribonucleoside diphosphate metabolic process	0.015 014 34
GO:0030099	myeloid cell differentiation	0.017 034 24
GO:0009185	ribonucleoside diphosphate metabolic process	0.017 637 24
GO:0043010	camera-type eye development	0.019 657 65
GO:0030326	embryonic limb morphogenesis	0.022 765 28
GO:0035113	embryonic appendage morphogenesis	0.022 765 28
GO:0060765	regulation of androgen receptor signaling pathway	0.026 579 77
GO:0048545	response to steroid hormone	0.029 894 05
GO:0051817	modulation of process of other organism involved in symbiotic interaction	0.031 447 91
GO:0050808	synapse organization	0.031 447 91
GO:0022604	regulation of cell morphogenesis	0.031 672 56
GO:0006090	pyruvate metabolic process	0.032 810 75
GO:0009132	nucleoside diphosphate metabolic process	0.037 789 62
GO:0030520	intracellular estrogen receptor signaling pathway	0.044 636 1
Shared Molecular Functions (MFs)		
GO:0042393	histone binding	1.760 9E-14
GO:0003712	transcription coregulator activity	2.254 9E-14
GO:0003713	transcription coactivator activity	4.821 7E-09
GO:0140297	DNA-binding transcription factor binding	3.739 2E-07
GO:0070577	lysine-acetylated histone binding	2.821 1E-06
GO:0140033	acetylation-dependent protein binding	2.821 1E-06
GO:0061629	RNA polymerase II-specific DNA-binding transcription factor binding	0.000 182 96
GO:0003714	transcription corepressor activity	0.000 286 47
GO:0042054	histone methyltransferase activity	0.000 286 47
GO:0140030	modification-dependent protein binding	0.000 360 25
GO:0016922	nuclear receptor binding	0.000 510 54
GO:0035257	nuclear hormone receptor binding	0.000 510 54
GO:0042800	histone methyltransferase activity (H3-K4 specific)	0.000 559 99
GO:0051427	hormone receptor binding	0.001 829 32
GO:0035258	steroid hormone receptor binding	0.003 469 83
GO:0017056	structural constituent of nuclear pore	0.006 834 18
GO:0008022	protein C-terminus binding	0.007 115 83
GO:0008013	beta-catenin binding	0.012 258 54
GO:0031490	chromatin DNA binding	0.018 423 29
GO:0042974	retinoic acid receptor binding	0.026 783 21
GO:0030374	nuclear receptor transcription coactivator activity	0.030 846 88
Shared Cellular Components (CCs)		
GO:0005667	transcription regulator complex	1.246 3E-09
GO:1904949	ATPase complex	1.971 3E-05
GO:0070603	SWI/SNF superfamily-type complex	5.238 2E-05
GO:0000123	histone acetyltransferase complex	0.001 954 87
GO:0034708	methyltransferase complex	0.003 558 42
GO:0035097	histone methyltransferase complex	0.003 777 82
GO:0031248	protein acetyltransferase complex	0.004 010 52
GO:1902493	acetyltransferase complex	0.004 010 52
GO:0036464	cytoplasmic ribonucleoprotein granule	0.004 966 25
GO:0035770	ribonucleoprotein granule	0.007 385 82
GO:0000812	Swr1 complex	0.046 105 31
GO:0048188	Set1C/COMPASS complex	0.046 105 31
GO:0071006	U2-type catalytic step 1 spliceosome	0.046 105 31
GO:0071012	catalytic step 1 spliceosome	0.046 105 31

Next, the KEGG pathway analysis in Notch1^HIGH^ and Notch2^HIGH^ cells demonstrated that the underrepresented mRNAs in Notch1^HIGH^ vs Notch1^LOW^ cells were associated with pathways involved in *focal adhesion* and *adherens junctions* (Fig. 3g), suggesting a reduced ability to perform cell-substrate and cell-cell interactions, typical of aggressive cancer cells.^16^ Interestingly, while Notch2^HIGH^ cells showed enriched *lysosome* and *glycosaminoglycan degradation* pathways (Fig. 3h) vs Notch2^LOW^ cells, relevant for the metastatic process,^17,18^ they displayed various downregulated pathways, including *nucleocytoplasmic transport*, *spliceosome*, *hepatocellular carcinoma*, *ubiquitin-mediated proteolysis*, *cell cycle*, *lysine degradation*, *mRNA surveillance* and *DNA replication* (Fig. 3i), compatible with the quiescent status associated with a possible cellular dormancy.^19–25^ Therefore, we propose that Notch2 rather than Notch1 affects the molecular machinery necessary for the quiescent status of a small subgroup of human BrCa MDA-MB231 cells.

### Stem cell signature and HSC mimicry

An important feature of dormancy is represented by the ability of metastatic cells to retain stem-like features.^18^ Furthermore, we had observed in our previous works that dormant cancer cells able to interact with SNOs expressed various stem cell markers.^11,12^ Therefore, we next asked whether the transcriptome analysis could unveil if Notch1^HIGH^ and Notch2^HIGH^ MDA-MB231 cells shared a pluripotency signature. Hence, Gene Set Enrichment Analysis (GSEA) was performed on our RNAdSeq datasets demonstrating that Notch1^HIGH^ cells showed no pluripotency signature compared to Notch1^LOW^ cells (Fig. 3j), while Notch2^HIGH^ cells displayed a clear-cut enrichment of pluripotency-associated transcripts (Fig. 3k) vs Notch2^LOW^ cells. These results support the hypothesis that Notch1 does not drive a stem-like phenotype in MDA-MB231 cells.

In our previous work, we also observed that Notch2^HIGH^ MDA-MB231 cells displayed similarities with the HSC population.^11^ Therefore, we asked whether Notch1^HIGH^ and Notch2^HIGH^ cells shared HSC signatures. GSEA results ruled out any HSC signature in Notch1^HIGH^ cells (Fig. 3l), while they showed a prominent HSC signature in Notch2^HIGH^ cells (Fig. 3m). Taken together, these data suggest that Notch2 rather than Notch1 cells display HSCs-like molecular phenotype.

### Role of HSC genes

**Fig. 4:**
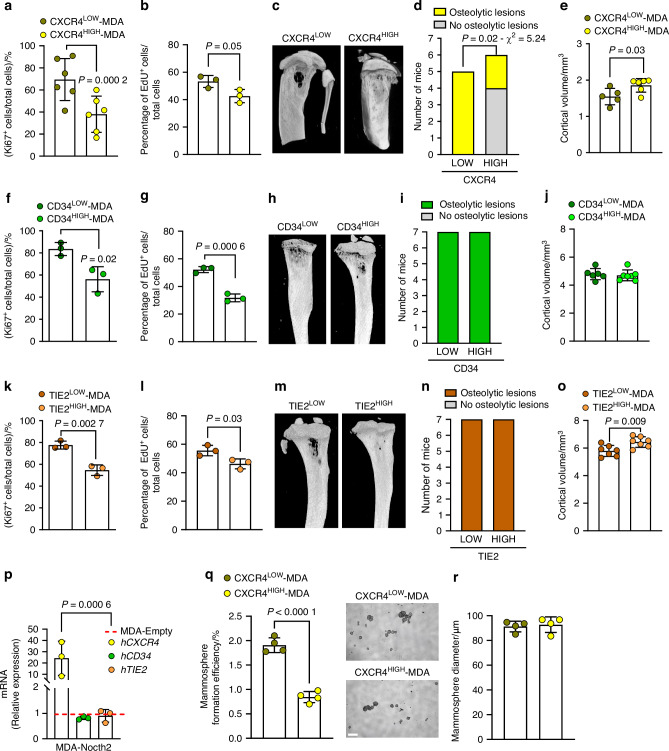
Role of HSC genes in BrCa progression. MDA-MB231 BrCa cells were MACS-sorted for LOW and HIGH expression of CXCR4, CD34 and TIE2 HSC genes. **a** Expression of the proliferation marker Ki67 and **b** incorporation of the thymidine analogue EdU in CXCR4^HIGH^ and CXCR4^LOW^ cells. **c** CXCR4^LOW^ and CXCR4^HIGH^ MDA-MB231 cells were intratibially injected in 4 weeks old CD1*nu*/*nu* immunocompromised mice. After 4 weeks, mice were sacrificed, and tibias were subjected to microCT analysis to identify osteolytic lesions. **d** Quantification of the number of mice developing osteolytic lesions and (**e**) of the tibia cortical bone volume in mice injected with CXCR4^LOW^ and CXCR4^HIGH^ MDA-MB231 cells. **f** Expression of the proliferation marker Ki67 and **g** incorporation of the thymidine analogue EdU in CD34^HIGH^ and CD34^LOW^ cells. **h** CD34^LOW^ and CD34^HIGH^ MDA-MB231 cells were intratibially injected in CD1*nu*/*nu* as described in **c** and subjected to microCT analysis to identify osteolytic lesions. **i** Quantification of the number of mice developing osteolytic lesions and **j** of the tibia cortical bone volume. **k** Expression of the proliferation marker Ki67 and **l** incorporation of the thymidine analogue EdU in TIE2^HIGH^ and TIE2^LOW^ cells. **m** TIE2^HIGH^ and TIE2^LOW^ MDA-MB231 cells were intratibially injected in CD1*nu*/*nu* mice as described in (**c**) and subjected to microCT analysis to identify osteolytic lesions. **n** Quantification of the number of mice developing osteolytic lesions and (**o**) of the tibia cortical bone volume. **q** Quantification of formation efficiency in CXCR4^HIGH^ and CXCR4^LOW^ cell mammosphere assay **r**. Measurement of mammosphere size in CXCR4^HIGH^ and CXCR4^LOW^ cell cultures (Bar = 150 µm). Results are the mean ± SD of 3–4 independent in vitro experiments or 5–7 mice/group; Statistics: unpaired *t* test (**a**–**o**, **q**, **r**) and multiple *t* test (**p**)

HSCs maintain their quiescence status through interactions with their respective niches, such as the endosteal niche.^8,9^ Our prior study demonstrated that Notch2^HIGH^ BrCa cells compete with HSCs for engraftment within the endosteal microenvironment.^11^ We examined the role of the HSC mimicry in MDA-MB231 cell cancer progression in bone, focusing on three HSC genes, *CXCR4*, *CD34*, and *TIE2*, that we previously found upregulated in MDA-MB231 dormant cells^12^ and that contributed to the HSC signature shown in Fig. 3m. MDA-MB231 cells were MACS-sorted for CXCR4, CD34, and TIE2 high and low expression and investigated for their ability to grow in vitro and generate bone tumours in vivo. CXCR4^HIGH^ cells were less positive to the proliferation marker Ki67 (Fig. 4a) and incorporated less 5-Ethynyl-2′-deoxyuridine (EdU) (Fig. 4b), suggesting an intrinsic lower proliferation ability. Consistently, when injected into the tibia of CD1 *nu*/*nu* immunocompromised mice, CXCR4^HIGH^ cells induced lesser extension (Fig. 4c) and incidence of osteolytic lesions as indicated by the lower number of mice presenting with tibia lytic areas (Fig. 4d) and their higher tibia cortical volume (Fig. 4e) compared to CXCR4^LOW^ cells.

Like CXCR4^HIGH^ cells, CD34^HIGH^ MDA-MB231 cells showed less positivity to the proliferation marker Ki67 (Fig. 4f) and incorporated less EdU (Fig. 4g). However, CD34^HIGH^ and CD34^LOW^ cells showed similar extension (Fig. 4h) and incidence (Fig. 4i) of osteolytic lesions and tibia cortical volume (Fig. 4j).

TIE2^HIGH^ MDA-MB231 cells exhibited lower proliferation ability in monolayers in vitro (Fig. 4k,l) and showed less in vivo extension (Fig. 4m) but equal incidence (Fig. 4n) of osteolytic lesion and more tibia cortical bone (Fig. 4o) compared to mice injected with TIE2^LOW^ cells.

Finally, the high and low expression of CXCR4 (Fig. S3a), CD34 (Fig. S3b) and TIE2 (Fig. S3c) did not change the expression of the cancer stem genes *CD24*, *CD44* and *ALDH1A2*, except for a slight increase of *CD24* in CD34^HIGH^ compared to CD34^LOW^ cells (Fig. S3b). Interestingly, the overexpression of the *NOTCH2* gene transfected in MDA-MB231 cells induced a higher expression of the *CXCR4* mRNA, with no impact on the expression of *CD34* and *TIE2* mRNAs (Fig. 4p). Additional evidence supporting the involvement of CXCR4 in MDA-MB231 cellular dormancy, was provided by a mammosphere assay, which demonstrated that CXCR4^HIGH^ cells generated fewer mammospheres than CXCR4^LOW^ cells (Fig. 4q), while the differences in mammosphere size were not significant (Fig. 4r). Altogether these results demonstrated that the expression of HSCs genes reduced the MDA-MB231 cell proliferation in vitro and their aggressiveness in vivo, except for the CD34. Moreover, overexpression data suggested a possible interaction between Notch2 and CXCR4 in MDA cells.

### ER stress signature

**Fig. 5:**
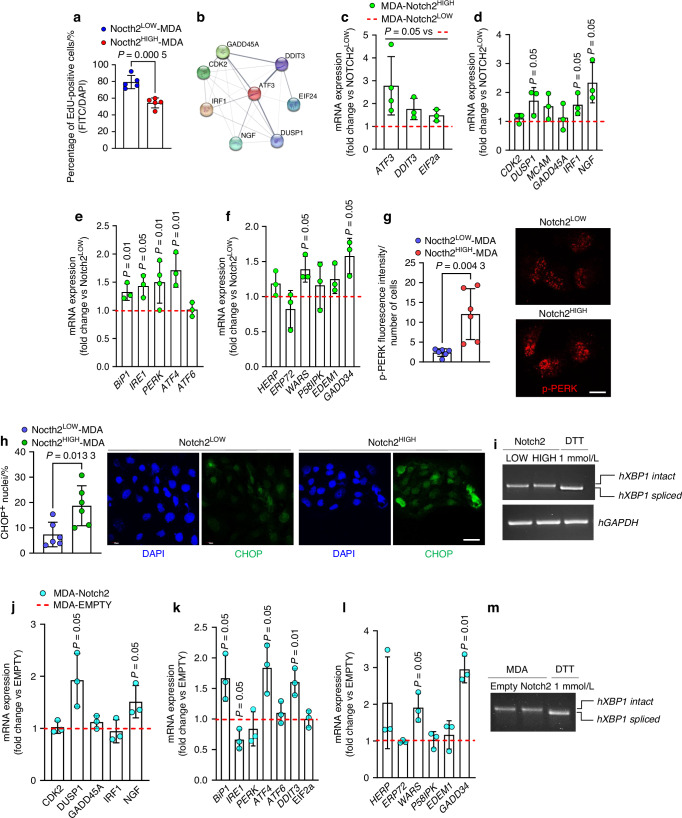
ER stress signature. **a** Incorporation of the thymidine analogue EdU in Notch2^LOW^ and Notch2^HIGH^ MDA-MB231 cells. **b** Notch2^HIGH^ MDA-MB231 cell ER stress and associated molecular network extrapolated from the RNAdSeq data. **c**–**f** Transcriptional expression of the indicated ER stress and associated genes in Notch2^HIGH^ vs Notch2^LOW^ MDA-MB231 cells. **g** Immunofluorescence detection of phosphorylated (p)-PERK protein in Notch2^LOW^ vs Notch2^HIGH^ MDA-MB231 cells (Bar= 10 µm). **h** Nuclear accumulation of CHOP protein in Notch2^LOW^ vs Notch2^HIGH^ MDA-MB231 cells (Bar = 15 µm). **i** Transcriptional expression of *hXBP1* in Notch2^LOW^ vs Notch2^HIGH^ MDA-MB231 cells. DTT (Dithiothreitol): *hXBP1* splicing positive control. **j**–**l** Transcriptional expression of the indicated ER stress and associated genes in Notch2-transfected vs empty vector-transfected MDA-MB231 cells. **m** Transcriptional expression of *hXBP1* in Notch2-transfected vs empty vector-transfected MDA-MB231 cells. DTT: *hXBP1* splicing positive control. Results are the mean ± SD of 3 independent experiments. Statistics: unpaired *t*-test

**Fig. 6:**
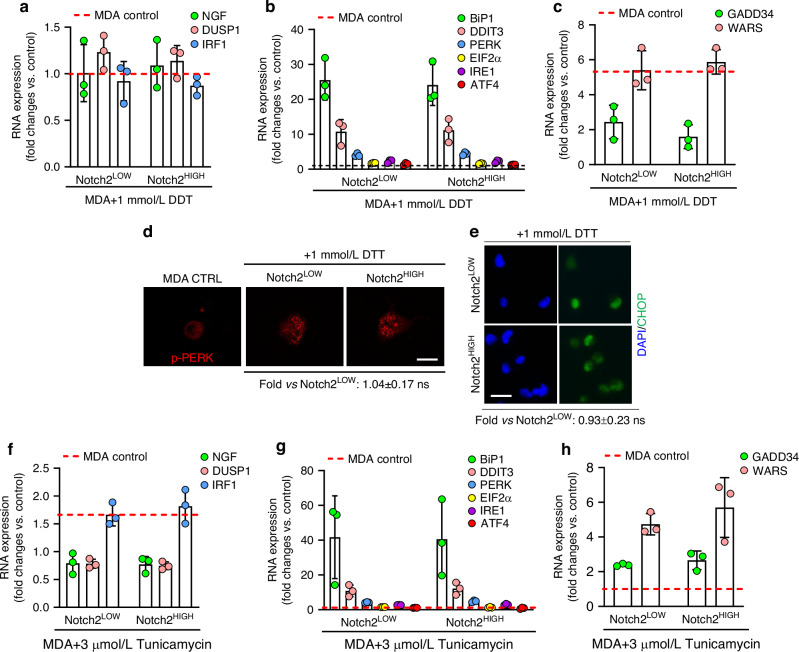
Induction of ER stress by DTT and Tunicamycin. **a**–**c** Transcriptional expression of the indicated ER stress and associated genes in Notch2^LOW^ and Notch2^HIGH^ MDA-MB231 cells treated with 1 mmol/L DTT. **d** p-PERK expression in control, Notch2^LOW^ and Nocth2^HIGH^ MDA-MB231 cells treated with 1 mmol/L DTT (Bar=10 µm). **e** Nuclear accumulation of CHOP in Notch2^LOW^ and Nocth2^HIGH^ MDA-MB231 cells treated with 1 mmol/L DTT (Bar=15 µm). **f**–**h** Transcriptional expression of the indicated ER stress and associated genes in Notch2^LOW^ and Notch2^HIGH^ MDA-MB231 cells treated with 3 μmol/L Tunicamycin. Results are the mean ± SD of 3 independent experiments. Statistics: unpaired *t*-test

ER stress has been implicated in various malignancies.^26–28^ Therefore, we aimed to investigate its potential role in the cellular dormancy of BrCa, focusing on Notch2^HIGH^ and Notch2^LOW^ MDA-MB231 cells. We first confirmed that Notch2^HIGH^ MDA-MB231 cells showed an intrinsic lower ability to incorporate the thymidine analogue EdU compared to Notch2^LOW^ cells (Fig. 5a), indicative of decreased proliferation, a characteristic potentially conferring protection against ER stress.^29^ Subsequently, we derived the ER stress-associated molecular network from RNAdSeq analysis and compared it between Notch2^HIGH^ and Notch2^LOW^ MDA-MB231 cells (Fig. 5b). We observed a higher expression of the ER associated *ATF3*, *DDIT3*, *EIF2α*, *DUSP1*, *IRF1* and *NGF* mRNAs in Notch2^HIGH^ cells compared to the low counterpart (Fig. 5c, d), along with a higher expression of the canonical ER stress markers *BIP1*, *IRE1*, *PERK* and *ATF4*, and the Unfolded Protein Response (UPR) genes, *WARS* and *GADD34* (Fig. 5e, f). In line with these results, immunofluorescence analysis revealed an increased PERK phosphorylation (Fig. 5g, Fig. S4) and CHOP nuclear translocation (Fig. 5h, Fig. S5) in the Notch2^HIGH^ compared to Nocth2^LOW^ MDA-MB231 cells. In contrast, no *XBP1* splicing was observed (Fig. 5i). Similar results were obtained in Notch2-transfected MDA-MB231 cells (Fig. 5j–m). Finally, the treatment with either dithiothreitol (DDT) or tunicamycin did not induce any further activation of ER stress in Notch2^HIGH^ vs Notch2^LOW^ MDA-MB231 cells (Fig. 6a–h), suggesting that the untreated Notch2-transfected cells had already achieved their maximal response. Overall, these data demonstrated the activation of an ER stress-associated molecular network in the Notch2^HIGH^ and Notch2-transfected MDA-MB231 cells, associated with the PERK pathway.

### Breast cancer cell-SNO interactome

**Fig. 7:**
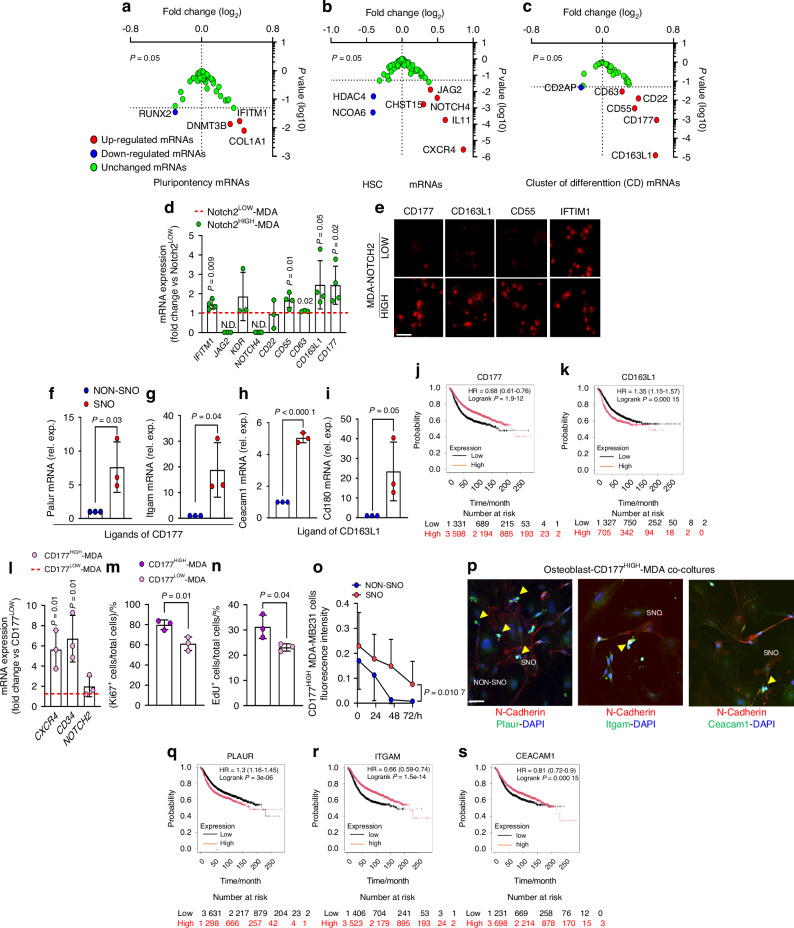
BrCa cell-SNO interactome. **a** Plots illustrating up-regulated, down-regulated and unchanged pluripotency, **b** HSC and **c** cluster of differentiation mRNAs in Notch2^HIGH^ vs Notch2^LOW^ MDA-MB231 cells. **d** Transcriptional expression of genes extrapolated from panels **a**–**c** encoding for cell surface proteins potentially involved in the cell-cell interaction between Notch2^HIGH^ cells and SNOs. **e** Protein expression in Notch2^HIGH^ and Notch2^LOW^ MDA-MB231 cells of the genes confirmed to be upregulated in **d** (Bar=20 µm). **f–h** Transcriptional expression in NON-SNO and SNO cells of potential CD177 and **i** CD163L1 ligands. NON-SNO values were normalised to 1 and SDs of the original unnormalized data were incorporated in the statistical analysis. **j** Kaplan-Meier plot in a cohort of BrCa patients with CD177 and **k** CD163L1 HIGH and LOW expression. **l** Expression of CXCR4, CD34 and Notch2 in MDA-MB231 cells MACS-sorted for CD177^HIGH^ and CD177^LOW^ expression. **m** Expression of the proliferation marker Ki67 and **n** incorporation of the thymidine analogue EdU in CD177^HIGH^ and CD177^LOW^ MDA-MB231 cells. **o** Proliferation assay performed by preincubation of CD177^HIGH^ cells with fluorescent cell tracer dye prior to their co-culture on SNO and NON-SNO monolayers (fluorescence intracellular retention associated with lower proliferation rate). **p** Immunofluorescence for the indicated proteins in osteoblast-CD177^HIGH^ MDA-MB231 cell co-cultures (arrows: CD177^HIGH^ MDA-MB231 cells located on N-Cadherin-positive osteoblasts (SNOs) (Bar = µm). **q** Kaplan-Meier plot in cohorts of BrCa patients with PLAUR, **r** ITGAM and **s** CEACAM1 HIGH and LOW expression. Results are representative of the mean ± SD of 3-4 independent experiments. Statistics: unpaired *t* test

According to the complexity of BrCa dormancy process in the endosteal niche, we took advantage of our Notch2 RNA dataset to identify new determinants mediating this interaction. Indeed, the RNAdSeq analysis unveiled several transcripts differentially expressed in Notch2^HIGH^ vs Notch2^LOW^ MDA-MB231 cells (Fig. S2 and Supplementary Data file 2). From this list we extracted the data relative to the most regulated mRNAs and focused on the transcripts associated with pluripotency, HSC signatures and Cluster of Differentiation (CD) (Fig. 7a–c and Table 3). From this list we selected mRNAs encoding for cell surface proteins, including *IFTM1*, *JAG2*, *KDR*, *NOTCH4*, *CD22*, *CD55*, *CD63*, *CD163L1* and *CD177*, that could be potentially involved in the interaction with SNOs.

**Table 3:** Transcript associated with pluripotency and HSC signatures and Cluster of Differentiation (CD) in Notch2^HIGH^ cells

Gene	Fold Change (Log_2_)	*P*-value
Pluripotency mRNAs		
CD34	0.268 632 7	0.090 380 7
CD9	0.294 211 41	0.091 318 64
CDH5	0.037 386 49	0.560 053 95
CDX2	n.a.	n.a.
CGB	−0.025 835 7	0.460 011 92
COL1A1	0.478 086 28	**0.007 914 33**
COL2A1	n.a.	n.a.
COMMD3	0.119 894 38	0.486 502 05
CRABP2	0.361 086 18	**0.049 623 54**
CTNNB1	0.054 332 97	0.532 914 77
DDX4	−0.010 397 4	0.821 645 74
DES	0.004 170 11	0.942 265 89
DNMT3B	0.321 499 81	**0.013 421 07**
EEF1A1	0.015 389 95	0.911 757 14
EOMES	0.004 942 76	0.933 515 91
FGF4	n.a.	n.a.
FGF5	−0.297 210 2	0.057 833 26
FLT1	0.046 748	0.397 866 85
FN1	0.130 813 01	0.444 813 68
FOXA2	−0.094 146 4	0.544 283 38
FOXD3	−0.040 867 4	0.764 879 52
GABRB3	0.018 355 55	0.523 853 77
GATA4	n.a.	n.a.
GATA6	0.195 947 05	0.271 732 96
GBX2	n.a.	n.a.
GCG	n.a.	n.a.
GCM1	0.012 207 37	0.754 073 46
GDF3	0.018 650 89	0.520 666 74
GFAP	0.061 587 36	0.236 797 9
GRB7	0.060 058 08	0.684 164 6
HBB	0.022 254	0.484 552 04
HBZ	n.a.	n.a.
IAPP	n.a.	n.a.
IFITM1	0.428 129 06	**0.017 075 12**
IFITM2	0.164 709 81	0.189 130 11
IL6ST	0.023 537 3	0.864 107 5
INS	n.a.	n.a.
ISL1	0.018 650 89	0.520 666 74
KIT	−0.012 110 9	0.790 568 51
KRT1	n.a.	n.a.
LAMA1	0.009 313 58	0.916 487 89
LAMB1	−0.130 639	0.423 120 39
LAMC1	−0.037 114 9	0.823 374 46
LIFR	−0.089 344	0.556 017 49
MYF5	n.a.	n.a.
MYOD1	n.a.	n.a.
NES	0.304 376 46	0.098 088 87
NEUROD1	n.a.	n.a.
NODAL	n.a.	n.a.
NOG	0.112 393 07	0.491 714 3
NPPA	n.a.	n.a.
NR5A2	0.070 980 61	0.646 378 81
NR6A1	0.059 813 93	0.722 340 5
OLIG2	n.a.	n.a.
PAX4	0.018 650 89	0.520 666 74
PAX6	n.a.	n.a.
PAX5	0.006 723 36	0.905 943 19
PODXL	0.029 934 34	0.825 779 78
POU5F1	0.027 453 63	0.679 122 94
PTEN	−0.025 097 9	0.771 999 13
PTF1A	n.a.	n.a.
RAF1	−0.006 934 3	0.961 102 06
REST	−0.213 279 2	0.100 440 34
RUNX2	−0.296 177 8	0.035 099 98
SEMA3A	−0.233 218 2	0.180 344 33
SERPINA1	0.133 768 34	0.358 735 72
SFRP2	n.a.	n.a.
SOX17	n.a.	n.a.
SOX2	0.015 469 39	0.912 736 15
SST	n.a.	n.a.
SYCP3	−0.047 761 6	0.448 114 91
SYP	0.110 490 72	0.523 888 5
TAT	n.a.	n.a.
TDGF1	−0.017 074 7	0.550 198 87
TERT	0.018 748 13	0.918 140 77
TFCP2L1	0.060 957 29	0.739 165 43
na		
UTF1	n.a.	n.a.
WT1	0.214 296	0.130 089 15
ZFP42	n.a.	n.a.
**HSC mRNAs**		
ANGPT1	0.090 815 73	0.616 637 12
APC	−0.177 264 4	0.277 406 88
ASH2L	−0.188 882 4	0.055 646 86
BLNK	−0.017 074 7	0.550 198 87
CBFB	−0.054 144	0.717 695 15
CCR1	−0.160 926	0.378 564 19
CD14	0.135 796 23	0.381 431 43
CD164	−0.000 377 1	0.997 092 91
CD1D	n.a.	n.a.
CD2	0.018 650 89	0.520 666 74
CD27	0.052 588 62	0.443 579
CD34	0.268 632 7	0.090 380 7
CD3D	0.018 650 89	0.520 666 74
CD3G	n.a.	n.a.
CD4	n.a.	n.a.
CD44	−0.008 226 2	0.933 327 44
CD80	0.006 509 13	0.908 993 52
CD86	n.a.	n.a.
CD8A	0.281 750 09	0.116 255 91
CEBPE	0.001 099 19	0.974 315 76
CEBPG	−0.054 101 5	0.739 743 64
CHST15	0.301 834 5	**0.001 660 55**
CSF1	0.239 520 9	0.193 072 68
CSF2	0.160 000 74	0.176 977 88
DLL1	−0.022 012 4	0.659 207 35
ETS1	0.049 530 94	n.a.
ETV6	0.014 712 21	0.930 648 09
FLT3LG	−0.166 349 1	0.337 029 3
FUT10	−0.154 473 1	0.310 675 85
FZD1	−0.156 111 6	0.341 180 22
GATA1	n.a.	n.a.
GATA2	0.148 838 15	0.260 529 58
HDAC4	−0.403 168	**0.005 087 04**
HDAC5	0.018 693 73	0.919 021 47
HDAC7	0.129 182 7	0.214 826 08
HDAC9	0.149 682 69	0.407 598 96
IL10	n.a.	n.a.
IL11	0.607 461 94	**0.000 180 48**
IL12B	0.031 481 77	0.544 323 77
IL1A	0.047 312 38	0.666 986 91
IL2	n.a.	n.a.
IL20	0.018 355 55	0.523 853 77
IL25	n.a.	n.a.
IL31RA	−0.256 363 8	0.112 027 8
IL6ST	0.023 537 3	0.864 107 5
INHA	0.200 997 67	0.182 717 1
INHBA	0.205 811 48	0.220 271 09
JAG1	0.000 301	0.998 624 41
JAG2	0.400 100 38	**0.012 985 59**
KDR	0.376 682 84	**0.038 223 4**
KIT	−0.012 110 9	0.790 568 51
KITLG	−0.186 874 7	0.306 466 87
LEF1	−0.317 541 7	**0.046 414 86**
LMO2	0.325 043 04	0.071 064 68
LRMP	0.022 700 76	0.696 030 37
MAL	0.018 068 2	0.526 992 52
MAP4K1	0.053 367 36	0.596 282 84
MMP9	0.097 365 83	0.340 179 06
NCOA6	−0.409 316 6	**0.000 519 74**
NOS2	0.000 262 7	0.993 811 76
NOTCH1	−0.150 175 8	0.167 646 42
NOTCH2	0.005 054 14	0.964 428 46
NOTCH4	0.495 032 21	**0.004 049 29**
PAX5	0.006 723 37	0.905 943 2
PECAM1	−0.000 641 2	0.996 597 68
PF4	−0.056 725 8	0.353 278 6
PTPRC	0.013 754 82	0.715 548 95
RBPJ	−0.110 968 2	0.261 548 73
RUNX1	−0.205 766 7	0.088 881 97
SFXN1	−0.106 781 5	0.445 310 43
SOCS5	−0.071 167 6	0.570 156 45
SPP1	−0.041 277 6	0.630 582 89
STAT1	−0.024 922 7	0.851 519 18
STAT3	0.076 654 6	0.508 923 14
STIM2	−0.133 296 1	0.439 107 85
TAL1	n.a.	n.a.
TEK	0.120 594 71	0.122 378 44
TLR3	0.164 238 1	0.369 555 92
TLR4	0.265 704 67	0.141 127 32
TNFSF11	n.a.	n.a.
TRIM10	0.032 253 99	0.397 175 43
VAV1	−0.059 745 7	0.405 006 58
VEGFA	−0.003 527 4	0.984 665 14
WNT3A	0.032 253 99	0.397 175 43
CXCR4	0.862 165 25	**2.77E-06**
**Cluster of Differentiation (CD) mRNAs**		
CD160	0.022 254	0.484 552 04
CD163	0.019 880 91	0.659 763 21
CD164L2	0.018 068 2	0.526 992 52
CD180	0.362 754 02	**0.003 331 08**
CD19	n.a.	n.a.
CD1A	n.a.	n.a.
CD1B	n.a.	n.a.
CD1C	n.a.	n.a.
CD1D	n.a.	n.a.
CD1E	n.a.	n.a.
CD2	0.018 650 89	0.520 666 74
CD200	n.a.	n.a.
CD200R1	0.001 099 18	0.974 315 76
CD200R1L	n.a.	n.a.
CD207	0.008 560 85	0.924 428 53
CD209	n.a.	n.a.
CD226	−0.034 430 1	0.762 393 86
CD244	n.a.	n.a.
CD247	n.a.	n.a.
CD248	n.a.	n.a.
CD27	0.052 588 62	0.443 579
CD28	0.022 685 57	0.818 078 42
CD300A	n.a.	n.a.
CD300C	0.021 992 27	0.487 022 97
CD300E	n.a.	n.a.
CD300LB	n.a.	n.a.
CD300LD	n.a.	n.a.
CD300LF	n.a.	n.a.
CD300LG	n.a.	n.a.
CD302	−0.054 312 1	0.538 839 65
CD36	0.029 270 47	0.664 387 83
CD37	0.018 068 2	0.526 992 52
CD38	0.114 955 31	0.143 334 13
CD3D	0.018 650 89	0.520 666 74
CD3E	−0.015 841 7	0.565 793 3
CD3G	n.a.	n.a.
CD4	n.a.	n.a.
CD40LG	n.a.	n.a.
CD48	n.a.	n.a.
CD5	0.022 250 76	0.604 850 72
CD52	0.095 469 04	0.192 613 54
CD53	0.012 207 37	0.754 073 46
CD5L	n.a.	n.a.
CD6	0.017 560 89	0.708 054 5
CD69	0.061 587 36	0.236 797 9
CD7	0.018 068 2	0.526 992 52
CD70	−0.009 698 4	0.802 070 46
CD72	0.068 620 83	0.531 946 12
CD79A	0.082 294 21	0.338 406 45
CD79B	0.136 158 25	0.073 172 67
CD80	0.006 509 13	0.908 993 52
CD81-AS1	−0.015 054	0.880 805 84
CD84	0.018 068 2	0.526 992 52
CD86	n.a.	n.a.
CD8B	n.a.	n.a.
CD93	−0.017 155 7	0.549 197 81
CD14	0.135 796 23	0.381 431 43
CD151	0.069 669 93	0.613 413 95
CD163L1	0.595 844 86	**1.27E-05**
CD164	−0.000 377 1	0.997 092 91
CD177	0.608 862 15	**0.000 913 49**
CD22	0.404 494 9	**0.012 459 96**
CD24	0.257 264 09	0.158 027 53
CD27-AS1	0.238 206 16	0.191 763 62
CD274	−0.219 459 8	0.057 117 65
CD276	0.056 883 92	0.748 432 84
CD2AP	−0.241 648 7	**0.047 540 02**
CD2BP2	0.054 289 76	0.685 252 35
CD320	0.059 435 73	0.662 777 46
CD33	−0.024 176 5	0.888 672 21
CD34	0.268 632 7	0.090 380 7
CD3EAP	−0.209 088 6	0.243 082 81
CD40	0.300 569 36	0.067 665 46
CD44	−0.008 226 2	0.933 327 44
CD46	0.054 956 97	0.717 530 08
CD47	0.124 823 78	0.302 368 38
CD55	0.362 159 57	**0.003 750 4**
CD58	−0.019 019 5	0.916 222 12
CD59	−0.037 389 1	0.806 026 9
CD63	0.220 736 54	**0.028 868 53**
CD68	0.038 813 29	0.804 795 27
CD74	0.179 959 6	0.283 309 74
CD81	0.080 578 76	0.350 046 97
CD82	0.147 852 77	0.270 388 67
CD83	−0.051 241 2	0.776 106 12
CD8A	0.281 750 09	0.116 255 91
CD9	0.294 211 41	0.091 318 64
CD96	−0.076 918 4	0.604 813 17
CD99	0.120 724 05	0.220 468 28
CD99L2	0.082 584 83	0.559 754 76
CD99P1	0.096 023 82	0.601 725 44

*n.a.* not available; **Bold:** statistically significant

We then subjected the Notch2^HIGH^ and Notch2^LOW^ cells to conventional real time RT-PCR to validate the differential expression of the selected genes. Results demonstrated that *CD177*, *CD163L1*, *CD55* and *IFITM1* mRNAs were significantly upregulated in Notch2^HIGH^ vs Notch2^LOW^ cells, whereas changes in *CD22* and *KDR* genes were not confirmed (Fig. 7d). Finally, although significant, the expression of *CD63* in Notch2^HIGH^ was only 1.1-fold higher than in Notch2^LOW^ cells, whereas the expression of *JAG2* and *NOTCH4* was neglectable in both groups (Fig. 7d).

Based on these results we focused on *CD177*, *CD163L1*, *CD55* and *IFITM1* genes and investigated the differential expression of their encoded proteins by immunofluorescence in Notch2^HIGH^ and Notch2^LOW^ MDA-MB231 cells (Fig. 7e). We observed that CD177, CD163L1 and CD55 proteins were more expressed in Notch2^HIGH^ than in Notch2^LOW^ cells, while IFTIM1 was equally expressed in the two groups (Fig. 7e).

We then excluded from the subsequent analysis the IFTIM1 protein, whose differential expression between Notch2^HIGH^ and Notch2^LOW^ cells was not confirmed, and used the String analysis to identify the known cell surface ligands of the CD177 (Fig. S6a), CD163L1 (Fig. S6b) and CD55 (Fig. S6c) encoded proteins, that could be expressed by SNO and NON-SNO cells. From this list, we extrapolated the cell surface proteins with an intracellular signal transduction activity (Fig. S6a–c) and evaluated the transcriptomic expression of their genes in SNOs and NON-SNOs. We found that the genes encoding the CD177 ligands Plaur (Fig. 7f), Itgam (Fig. 7g), and Ceacam1 (Fig. 7h), and the gene encoding the CD163L1 ligand Cd180 (Fig. 7i) were expressed several folds more in SNOs vs NON-SNOs, whereas all other identified ligands were downregulated or not expressed in SNOs vs NON-SNOs (Fig. S6d–h).

To provide a translation meaning to these observations, we investigated if *CD177*, *CD163L1* and *CD55* correlate with the severity of human BrCa disease. To this purpose, we interrogated the Kaplan–Meier plots (KMPlot®) database containing 4929 public transcriptomes from primary BrCa. We observed that there was a statistically positive correlation in Kaplan-Meyer diagrams between the high expression of *CD177* and the overall survival of patients (Fig. 7j), whereas this correlation was negative when the *CD163L1* (Fig. 7k) and *CD55* (Fig. S6i) transcriptomes were interrogated. Furthermore, overexpression of Notch2 induced an increase of *CD177* expression in MDA-MB231 cells, while *CD163L1* and *CD55* expressions were unremarkable (Fig. S6j), further suggesting an association only between Notch2 and CD177. Finally, given the poor expression in SNOs of the genes encoding for the CD163L1 and CD55 protein ligands and the positive correlation in the Meyer-Kaplan diagrams observed only for the CD177 expression in cancer cells, we focused on the protein encoded by this gene and sorted MDA-MB231 cells for CD177^HIGH^ and CD177^LOW^ expression, analysing whether there were phenotypic and functional similarities between them and Notch2^HIGH^ and Notch2^LOW^ cells. Results demonstrated that CD177^HIGH^ cells were also CXCR4^HIGH^, CD34^HIGH^ and Notch2^HIGH^ (Fig. 7l) and proliferated less than CD177^LOW^ cells (Fig. 7m, n). Interestingly, CD177^HIGH^ cells exhibited reduced proliferation when cultured on SNOs compared to NON-SNOs (Fig. 7o), suggesting that interactions with SNOs impaired their doubling ability.

To investigate the role of CD177 and its associated SNO cell ligands in cellular dormancy, an in vitro functional assay was conducted in which CD177^HIGH^ cells were labelled with Violet 450 proliferation tracer and cultured on SNO and NON-SNO monolayers. Proliferation was quantified by measuring the fluorescence intensity of CD177^HIGH^ cells, with fluorescence diminishing over time because of cell division. The results demonstrated enhanced proliferation of CD177 ^HIGH^ cells plated on NON-SNOs, as shown with the fluorescent dye reaching near baseline in 48 hours (Fig. 7o). In contrast, CD177^HIGH^ cells plated on SNOs retained higher fluorescence levels up to 72 h, reflecting reduced proliferative activity and greater dye retention (Fig. 7o).

We then investigated where Plaur, Itgam, and Ceacam1 proteins were located in osteoblast-CD177^HIGH^ MDA-MB231 cell co-cultures. As shown in Fig. S7, SNOs (N-Cadherin^HIGH^ osteoblasts) expressed Plaur, Itgam, and Ceacam1, consistent with expectations, whereas NON-SNOs (N-Cadherin^LOW^ osteoblasts) lacked these proteins. Notably, CD177^HIGH^ MDA-MB231 cells tended to be associated with SNOs (Fig. 7p), implying specific interaction, potentially mediated by binding between CD177 on MDA-MB231 cell and the Plaur, Itgam, and Ceacam1 present on SNOs. Furthermore, we discovered that the CD177^HIGH^ MDA-MB231 cells themselves expressed the human proteins PLAUR, ITGAM, and CEACAM1 (Fig. S7), adding further complexity to these cellular interactions.

To thoroughly assess the potential translational significance of our observations, we utilized the Kaplan-Meier platform to examine the relationships between PLAUR, ITGAM, and CEACAM1 expression in BrCa and patient prognosis. The analysis indicated that, surprisingly, elevated PLAUR expression (Fig. 7q) was associated with poorer outcomes, whereas increased ITGAM (Fig. 7r) and CEACAM1 (Fig. 7s) expression were significantly linked to more favourable prognoses. These findings suggest meaningful translational relevance, but at the same time underscore distinct differences emphasizing the need for further studies to clarify their roles in cellular dormancy.

## Discussion

This work was inspired by our previous results showing dormancy features in Notch2^HIGH^ BrCa cells when they interacted with SNOs.^11^ In that study we also demonstrated that Notch3 and Notch4 were irrelevant for this feature, while Notch1 appeared to be less prominent in cell dormancy, although we could not definitely draw conclusions on this aspect. Here we used an integrated strategy involving morphology, cell biology, RNAdSeq, molecular biology and in vivo studies that allowed us to mitigate the role for Notch1 in this context. In fact, we observed paucity of Notch2^HIGH^ cells in samples of human BrCa tissues compared to Notch1^HIGH^ cells, especially in moderately and poorly differentiated cancers. Notch2^HIGH^ cell rareness was even more evident in the bone metastasis we could retrieve from our archive. Unfortunately, the lack of other metastasis samples prevented us from performing a reliable computation of the Notch2/Notch1 ratio in this tissue. However, despite this limitation, our observations appear to be in line with the dormancy phenotype given that only a minority of metastatic cells are known to persist in the colonized organs in a quiescent status even for decades.^19^ Intriguingly, >50% of Nocth2^HIGH^ cells were also Notch1^HIGH^, representing a confounding element to understand which of the two Notches was prominent in our conditions of cellular dormancy. This doubt was resolved investigating T47D cells sorted for Notch1^HIGH^, which were Notch2-negative and showed no proliferation changes when plated on SNO vs NON-SNO cells, suggesting that Notch1 is moderately implicated in SNO-induced dormancy. This hypothesis is also supported by our previous result^11^ obtained silencing individually Notch1 and Notch2 in MDA-MB-231 cells by specific shRNAs. Analysis after seeding on SNOs demonstrated that the rescue of proliferation was two third more prominent in Notch2- compared to Notch1-silenced cells.^11^

Consistently, KEGG analysis showed that only Notch2^HIGH^ cells displayed pathways whose increment is associated with metastatic features, and pathways whose decrement is associated with the ability to maintain a quiescent status. In fact, enrichments of *lysosome* and *glycosaminoglycan degradation* pathways have been found to be involved in changes stimulating the metastatic process and are now being investigated to improve therapies.^18,21–25,30,31^ On the other hand, many pathways downregulated in Notch2^HIGH^ cells are known to interfere with events relevant for cancer spreading, including *nucleocytoplasmic transport*,^32^ *spliceosome*,^33^ *hepatocellular carcinoma*,^34^ *ubiquitin-mediated proteolysis*,^35^ *cell cycle*,^36^ *lysine degradation*,^37^ *mRNA surveillance*^38^ and *DNA replication*.^39^ This series of molecular observations supported a role for Notch2 in the maintenance of a quiescent status in bone metastases.

In agreement with our previous observations showing competition of Notch2^HIGH^ BrCa cells with the HSC lineage for the engraftment of the endosteal niche,^11^ in this study we documented that Notch2^HIGH^ cells showed pluripotency and HSC-like gene signatures, which were not observed in Notch1^HIGH^ cells. SNOs are known to contribute to HSC quiescence.^8^ In support of the relevance of Notch2^HIGH^ BrCa cells in sharing stem features with HSCs we demonstrated that, like HSCs, Notch2^HIGH^ cells were also CXCR4^HIGH^, CD34^HIGH^ and TIE2^HIGH^.^11^ CXCR4 is a chemokine receptor binding CXCL12 with intricated roles in cancer.^40^ It triggers various pathways involved in cell migration, cell homing, haematopoiesis and retention in the bone marrow.^41^ It is highly expressed in several tumours, including BrCa, correlating with poor prognosis.^42^ Interestingly, CXCR4 is also expressed by HSCs, contributing to their maintenance in the bone marrow stromal cell niche,^43^ which includes endosteal cells.^44,45^ CD34 is instead a transmembrane phosphoglycoprotein expressed by HSCs, implicated in many cell functions, including cell-cell adhesion.^46^ CD34^+^ HSCs have recently been observed to display high repopulating and self-renewal abilities, leading to their multilineage output.^47^ TIE2 is a receptor binding angiopietin-1, maintaining HSC activity, which is antagonized by angiopoietin-2, another TIE2 ligand.^48^

The high expression of these HSC genes in MDA-MB231 cells seems to be associated with different biological functions. They converged in showing a lower proliferation rate in vitro but, while CXCR4^HIGH^ cells were less prominent in inducing in vivo osteolytic lesions compared to CXCR4^LOW^ cells, TIE2^HIGH^ cells induced in vivo equal number but smaller osteolytic lesions and CD34^HIGH^ cells showed no changes in osteolytic lesion incidence and size compared to their LOW counterpart. Intriguingly, we observed that CXCR4 levels rise in MDA-MB231 cells when Notch2 is overexpressed, consistent with reports of high Notch2 and CXCR4 expression in quiescent bone marrow HSCs.^49^ Furthermore, other studies showed that mitotic quiescence in prostate cancer also depends on high CXCR4, supporting our findings in BrCa.^50^ While some research links high CXCR4 and its ligand CXCL12 to increased cancer cell survival, proliferation, angiogenesis, and metastasis—especially in bone—,^45^ others suggest overexpressed CXCR4 mainly aids bone marrow niche colonization and cellular dormancy maintenance rather than induction.^51–53^ Unlike CD34^LOW^, CD34^HIGH^ cells did not affect osteolytic lesion incidence or size, and, to the best of our knowledge, there is no evidence in literature that CD34 contributes to cancer cell dormancy. Finally, in BrCa patients, higher TIE2 expression correlates with delayed metastases and improved survival, and in vitro and mouse data further show TIE2 reduces BrCa proliferation and bone metastasis,^54^ thus supporting our results. However, given the complexity highlighted by several studies, further research is required to fully clarify the molecular mechanisms involved.

One limitation of these experiments was the use of immunocompromised mice injected intratibially with tumour cells, which does not fully replicate natural conditions. Nevertheless, this method enabled the investigation of human BrCa cells within an immunologically permissive environment and allowed for the injection of minimal cell numbers due to the rarity of dormancy. Moreover, our previous study,^11^ employing a similar model with mouse 4T1 cells injected intratibially into immunocompetent mice, produced results consistent with those observed for human MDA-MB231 cells in immunocompromised mice. Therefore, despite these limitations, we consider our findings to be robust and believe they provide a foundation for further research in this field.

ER stress plays a relevant role in cancer, and various cancer-induced anomalies disrupt ER homeostasis in both malignant and stromal cells.^27^ Therefore, we hypothesised that ER stress could be involved in cellular events inducing dormancy. In fact, we demonstrated that expression of ER canonical and associated genes responsible of the UPR response was upregulated in Notch2^HIGH^ compared to Notch2^LOW^ cells, and in Notch2-transfected cells vs controls. We also observed increased phosphorylation of PERK and nuclear translocation of CHOP in both groups of Notch2 overexpressing cells, in agreement with existing data.^55^ PERK represents a survival factor for dormant cancer cells and PERK suppressive therapy has been proposed to target growth arrested cells that escape anti-proliferative therapies.^56^ PERK and CHOP are members of the UPR family^57^ and they were consistently activated in our Notch2^HIGH^ vs Notch2^LOW^ cells. Indeed, we observed increased phosphorylation of PERK and nuclear translocation of CHOP in both groups of Notch2 overexpressing cells, in agreement with existing data.^58^ PERK is also known to contribute to cell-cycle arrest phosphorylating elF2α and to increment cell survival inducing ATF4.^58^ Consistently, in our conditions we found a strong increase of ATF4 expression in Notch2^HIGH^ vs Notch2^LOW^ cells and in Nocth2-trasfected vs control cells, while a less prominent increase of expression of elF2α was observed in sorted Notch2^HIGH^ vs Notch2^LOW^ cells, consistent with its role in cell-cycle arrest by phosphorylation rather than by induction.^58^ Finally, the lack of *XBP1* splicing in Notch2^HIGH^ and in Notch2-transfected cells is consistent with a lack of effect of this pathway in reducing cell survival.^59^

Finally, with a “large-to-narrow” approach we identify the CD177 as a gene potentially implicated in the SNO-induced Notch2^HIGH^ cellular dormancy. From a large group of transcripts overexpressed by Notch2^HIGH^, associated with pluripotency, HSC signature and cluster of differentiation, only CD177 was confirmed to be overexpressed in Notch2^HIGH^ cells at the RNA and protein level, while SNO cells expressed at least three CD177 ligands (Plaur, Itgam and Ceacam1). The CD177 overexpression correlated positively in Kaplan-Meyer diagrams with the overall survival of BrCa patients, was associated with high expression of the HSC genes CXCR4, CD34 and TIE2 and inhibited BrCa cell proliferation compared to CD177 low expression. CD177 is a glycosylphosphatidylinositol (GPI)-anchored protein expressed especially in neutrophils and involved in their cell migration. It binds β2 integrins,^60^ PR3^61^ and PECAM-1,^62^ promoting neutrophil migration,^63^ attenuates β-catenin signalling in tumours^63^ and regulates tumour-infiltrating T cells.^64^ To the best of our knowledge, CD177 has not yet been associated with tumour dormancy. However, it was associated with good prognosis in colorectal carcinoma,^65^ while it is expressed in normal breast tissue and reduced in invasive BrCa.^65^ Furthermore, patients with high numbers of CD177^+^ neutrophils show improved overall and disease-free survival.^65^ Likewise, in our study, higher expression of ITGAM and CEACAM1, but not PLAUR, also positively correlated with patients’ overall survival. ITGAM was found significantly lower in osteosarcoma tumour cells than in the normal bone,^66^ while its high expression positively correlated with a good prognosis in triple negative BrCa.^67^ CEACAM1 positively correlated with overall survival of basal-like BrCa and its mRNA expression was significantly lower in subgroup of basal-like BrCa with poorer prognosis.^68^ In contrast, high levels of PLAUR correlated with poor patient prognosis in BrCa.^69^ These data support our observations. However, further work is necessary to draw hypotheses on the role of these BrCa genes in cellular dormancy.

In conclusion, our work provided new information on the molecular pathways implicated in cellular quiescence, a condition that, upon environmental changes, induces cancer relapse after apparent cure. These pathways are multiple, redundant and intricated, requiring more effort and probably new methodologic approaches, including spatial multiplex and AI-assisted imaging, to be described in detail for the benefit of patients affected by BrCa and other types of malignancies.

## Materials and methods

### Materials

Plastic wares were from Falcon Becton-Dickinson. Ultralow attachment plates were from Corning (cat# CLS7007). Dulbecco’s Modified Eagle Medium (DMEM) (cat# ECB7501L), DMEM/F12 medium (Cat# ECM0095A10), Hank’s Balanced Salt Solution (HBBS) (cat# ECB4007L) and Phosphate Buffered Saline (PBS) (cat# ECB4004L) for cell culture were provided by EUROCLONE. Foetal Bovine Serum (FBS) (cat#26140-079), Lipofectamine 2000 (cat# 11668027), Trizol® reagent (cat# 15596026) and primers were from Invitrogen. Penicillin/streptomycin (cat# 15140130, and L-glutamine (cat# A2916801) were for Thermo Fisher Scientific. RevertAid H Minus First Strand cDNA Synthesis Kit (cat# K1631) and Ki-67 antibody (cat# MA5-14520) were from Thermo Scientific. ClickTech EdU cell proliferation kit(#BCK-EdU488HTS2) was from BaseClick DNA&RNA experts. b-FGF (cat# AF-100-18B) and EGF (cat# AF-100-15) were from Peprotech). B27 (cat# 17-504-044) and N2 (cat# 17-502-048) were from GIBCO. The Luna® Universal qPCR Master Mix (cat# M30303E) was from Bio Labs. RNAeasy mini (cat# 74104) was provided by Qiagen. Protease inhibitor cocktail (cat# P8340) was from Sigma Aldrich. All reagents for histology were from Bio-Optica. Cell Proliferation Tracer kit (#4844) and SignalStain® Boost IHC Detection Reagent (8114S anti-rabbit) were from Cell Signaling. All regents for magnetic-activated cell sorting (MACS) were from Miltenyi Biotec. Notch2 antibody (cat# Sc-5545) was from Santacruz Biotechnology. Breast cancer tissue array (cat# T8235721-5) was from BioChain®. Primary BrCa and matched metastasis were retrieved from archive material. Trypsin (cat# 85450 C) was from SAFC Biosciences. All other reagents were of the purest grade from Sigma Aldrich Co.

### Animals

The in vivo study was conducted in agreement with the national and international guidelines and policies (European Economic Community Council Directive 86/609, OJ L 358, 1, December 12, 1987; Italian Legislative Decree 4.03.2014, n.26, *Gazzetta Ufficiale della Repubblica Italiana* no. 61, March 4, 2014) and were approved by the Italian Ministry of Health (N.270/2018-PR; N.1551/2020-PR). Mice were humanely sacrificed by CO_2_ inhalation. The study was performed according to the Animal Research: Reporting of In Vivo Experiments (ARRIVE) guidelines (Table S5).

### Human samples

We used archive human samples from our biobank, and BrCa tissue array (cat# T8235721-5) purchased from BioChain®, containing 64 different primary breast tumours, plus positive and negative controls. Grade of differentiation, classification of primary tumour size (T), degree of spread to regional lymph nodes (N), presence of distant metastasis (M), and expression of oestrogen receptor (ER), progesterone receptor (PR) and receptor tyrosine-protein kinase erbB-2 (HER2) are illustrated in Table S1. Spare samples of BrCa and matched metastasis were retrieved from archive material, with no detailed information on patients’ status. Kaplan-Meier diagrams were constructed using public patient cohorts of 4 929 public transcriptomes from primary human breast cancers (KMPlot®, Kaplan-Meier plotter).

### Cell lines

The human BrCa cell lines MDA-MB231, T47D, ZR75D, BT474, and the murine BrCa cell line 4T1 were purchased from the American Type Culture Collection (ATCC) and cultured according to the supplier instruction. Cells were trypsinised and used for experiments as described in the specific sections.

### Primary osteoblast cell isolation

Murine osteoblasts were isolated from the calvarias of 7–10-day-old CD1 mice. Calvarias underwent 3 steps of incubation at 37 °C with a digestion solution containing trypsin (25 mg/mL) and clostridium histolyticum type IV collagenase (1 mg/mL) in Hanks’ Balanced Salt Solution. Cells from the second and third digestions were osteoblast enriched.

### Immunofluorescence and immunohistochemistry

Cells and tissue sections were labelled with primary antibodies (dilution 1:100–1:200) or stained with haematoxylin and eosin. For both cells and tissue samples, primary antibody incubations were carried out at room temperature for 1 h, then overnight at 4 °C, followed by incubations for 1 h at room temperature with the corresponding secondary antibody at dilution 1:500. Images were collected by an Olympus FV1200 confocal microscope (Tokyo, Japan).

### Magnetic-associated cell sorting (MACS)

Cells were detached and suspended in sterile sorting buffer containing 5% BSA and 0.5 mol/L EDTA in DPBS. Cell suspensions were incubated for 20 min at 4 °C with the primary antibody (3 μL/10^6^–10^7^ cells) against the surface protein of interest. Then cells were incubated in the same conditions with anti-PE antibody or streptavidin, respectively, conjugated to magnetic microbeads (20 μL/107 cells). Afterwards, cells were run through the magnetic column to obtain separate antigen-depleted and antigen-enriched cell populations.

### Mammosphere cultures

For mammosphere cultures, CRXC4^LOW^ and CXCR4^HIGH^ MDA-MB-231 cells were suspended at 4 000 cells/mL and seeded into ultralow attachment plates in serum free DMEM/F12 (1:1) supplemented with 10 ng/mL basic fibroblast growth factor, 20 ng/mL epidermal growth factor, 1% B27, 1% N2, 1% penicillin/streptomycin and 1% L-glutamine. Twenty % of fresh medium was added to each well every two days (without removing the old medium). The number of spheres (diameter >50 μm) for each well was evaluated using a Nikon Eclipse Ts2 microscope with a 4× objective at 5 days and analysed using the ImageJ software. Mammosphere Formation Efficiency % (MFE %) was calculated as the number of spheres in each well divided by the original number of cells seeded. The mammosphere diameter was also evaluated by the ImageJ software.

### Proliferation assays

Proliferation was evaluated by EdU incorporation assay or by cell proliferation cell tracer dilution assay, followed by fluorescence detection, according to the manufacturers’ instruction.

### Flow cytometry

Cells were detached and suspended in sterile sorting buffer containing 5% Bovine Serum Albumin (BSA) and 0.5 mol/L EDTA in DPBS. Cell suspensions were incubated for 1 h at 4 °C with 10 μL per 10^7^ cells of primary antibodies along with 3 μL per 10^7^ cells of fluorescent secondary antibody. Afterwards, cells were analysed using FACScantoII equipped with the FACSDiva software.

### Transcriptome analysis

Three independent RNA preparations of Notch1 and Notch2 HIGH and LOW cells were precipitated in ethanol and sent to GATC Biotech (Germany) for the RNAdSeq analysis.

### Transcriptome alignment, annotation, and differential expression analysis

RNAdSeq reads were aligned to the reference transcriptome (mm10/GRCm38, Ensembl; v85 Ensembl) by GACT. Transcripts were compared using Cuffdiff to determine the differential expression levels at the transcript level between samples. The generated RNAdSeq datasets, containing the expression profile of 36 000 genes for each sample/condition, were used for the Gene Ontology (GO) and Kyoto Encyclopedia of Genes and Genomes (KEGG) pathway enrichment analysis.^70^

### Gene ontology and pathway analyses

Normalized differential gene expression data generated by RNAdSeq was analysed by the R package, GOseq^70^ using an online database to remove any effect that gene length may have on the expression levels of some of them. The package then calculates the GO categories significantly enriched with differentially expressed genes. GO graphical representation was done using the free web-tool REVIGO.^71^

The R package GOseq was also used to identify the enrichment of differentially expressed genes in KEGG pathways.^70^ The package identifies enriched and unenriched KEGG pathways by calculating an adjusted p-value using the Wallenius non-central hypergeometric distribution.

### Real time RT-PCR

Total RNA was extracted using Trizol® reagent and quantified by Nanodrop. RNA quality was evaluated by 1% agarose gel run. For cDNA synthesis, 1 μg of RNA was reverse transcribed using the RevertAid H Minus First Strand cDNA Synthesis Kit. Real time PCR reaction was performed loading 0.1 μg of cDNA using the Luna® Universal qPCR Master Mix. Gene expression data were represented as fold change over the control and normalized by *Gapdh*, unless otherwise stated.

### In vivo studies

Mice included in the in vivo study were CD1 *nu*/*nu* strain, females, age 5 weeks, weight (15-30) ± (3-5) g. MDA-MB231 cells (1 × 10^5^/0.01 mL PBS) were injected into the left tibia of mice anaesthetised with intraperitoneal injection of 80 mg/kg of ketamine and 10 mg/kg of xylazine, using standard procedures. Group assignment was random. The attrition rate of the experiment was 9.5% (*n* = 4 mice), which is relatively low for a study involving cancer development. The procedure allowed intratibial growth of the tumours with no tumour protrusion outside the bone during the 4-week follow up.

Bias reduction for the analysis was achieved by blinding of investigators. Animals were monitored daily for body weight, behaviour, and survival. Weekly, mice were also subjected to deep anaesthesia and X-ray analysis (peak kilovoltage [kVp] = 36 kV for 10 s) using a Cabinet X-ray system (Faxitron model no.43855 A; Faxitron X-Ray Corp., Buffalo Grove, IL, USA) to follow the onset and progression of osteolytic lesions. At the end of the experiment, mice were euthanised and subjected to anatomical dissection for the evaluation of bone lesions.

### Micro-computed tomography

Images from tibias fixed in 4% paraformaldehyde were acquired in a SkyScan 1174 with a resolution of 6 μm (X-ray voltage 50 kV). Skyscan Nrecon software was used to employ the Feldkamp algorithm and reconstruct the images. Three- and two-dimensional (3D and 2D, respectively) morphometric parameters were calculated for the trabecular bone. Segmentation of the bone was conducted using threshold values corresponding to bone mineral density values of 0.6 cm^3^ calcium hydroxyapatite. Marching cube-type models with a rendered surface formed the basis of the 3D parameters. 2D areas were calculated using the Pratt algorithm. Bone structural variables and nomenclature were those suggested by Bouxsein et al.^72^

### Statistical analysis

Data from RNAdSeq analysis are representative of three mRNA datasets derived from 3 independent cultures. For the differential gene expression in Notch 1 and Notch2 HIGH and LOW cells, an uncorrected p-value generated by the Cuffdiff analysis was used. The statistical significance for the enrichment analyses was computed using a Benjamini-Hochberg adjusted *P* < 0.05. A *P*-value ≤ 0.05 was considered statistically significant. For the other experiments, the statistical analyses were carried out using the unpaired, two-tailed Student’s *t-*test with the software Prism® by GraphPad v7.0. *P*-values threshold was *<* 0.05.

## Supplementary information

Supplemental material

Supplemental Data file 1

Supplemental Data file 2

Supplemental Data file 3

Supplemental Data file 4

Supplemental Data file 5

Supplemental Data file 6

## Data Availability

All data presented within the article and its supplementary information files are available upon request from the corresponding author.
