# Supplementary material for: The endosteal niche regulates breast cancer cell dormancy in bone: identification of new molecular determinants
Source: Bone Res. 2026 May 14;14:51. doi: 10.1038/s41413-026-00535-3 (PMC13176332; doi:10.1038/s41413-026-00535-3)
Supplement: Supplementary file 1 — Supplemental material [file 41413_2026_535_MOESM1_ESM.pdf]

Supplementary Material

The endosteal niche regulates breast cancer cell dormancy in bone.

Identification of new molecular determinants.

Antonio Maurizi[1*], Maria Salbini[2*], Michela Ciocca[1], Marzia Rea[1], Giuseppe D. Tocchini-Valentini[2,3], Matilde Merolle[2], Hanna Taipaleenmäki[4,5], Christina Møller Andreasen[6], Manuela Pellegrini[2^] and Anna Teti[1,2^]

[1]Department of Biotechnological and Applied Clinical Sciences, University of L'Aquila, L'Aquila, Italy

[2]Institute of Biochemistry and Cell Biology, National Council of Research, Monterotondo (Rome), Italy

[3]European Mouse Mutant Archive (EMMA), INFRAFRONTIER-IMPC, Mouse Clinic, National Council of Research, Monterotondo (Rome), Italy.

[4]Institute of Musculoskeletal Medicine, LMU University Hospital, LMU Munich, D-82152 Planegg-Martinsried, Germany

[5]Musculoskeletal University Center Munich, LMU University Hospital, LMU Munich, D-82152 Planegg-Martinsried, Germany.

[6]Research Unit of Pathology, Department of Clinical Research, University of Southern Denmark, Odense, Denmark

Supplementary Table 1. Tissue array sample features

Patient #	Grade of differentiation	TNM1	ER	PR	Her2
**A3**	moderate	T2N2M1	-	-	++
**A4**	moderate	T2N2M1	-	-	-
**A5**	moderate	T2N1M0	+	-	-
**A6**	moderate	T2N2M1	+	+	-
**A7**	moderate	T2N1M1	-	-	-
**A8**	moderate	T2N0M0	-	-	-
**A9**	moderate	T2N0M0	+	++	-
**A10**	moderate	T2N0M0	++	+	-
**A11**	moderate	T2N2M1	+	-	-
**B1**	moderate	T2N0M0	+++	+++	-
**B2**	moderate	T2N0M0	-	-	-
**B3**	well	T2N2M0	-	-	-
**B4**	well	T2N2M0	+	+	-
**B5**	moderate	T2N1M0	+++	++	-
**B6**	moderate	T2N2M0	-	+++	-
**B7**	moderate	T2N1M1	-	-	-
**B8**	moderate	T2N1M1	-	-	-
**B9**	Poor	T3N3M0	++	+	-
**B10**	moderate	T2N1M0	-	-	-
**B11**	moderate	T2N2MO	-	-	-
**C1**	moderate	T2N1M0	++	+	-
**C2**	moderate	T2N0M0	-	-	-
**C3**	moderate	T2N1M1	+++	-	-
**C4**	moderate	T2N3M1	-	-	++
**C5**	moderate	T2N2M0	-	-	-
**C6**	Poor	T2N0M0	++	+	-
**C7**	Poor	T2N0M0	+	+	-
**C8**	N/A	T2N1M1	-	-	-
**C9**	N/A	T1N0M0	-	-	-
**C10**	N/A	T2N3M1	+	-	-
**C11**	N/A	T1N0M0	+	+	++
**D1**	N/A	T2N0M0	+	+	-
**D2**	moderate	T2N2M0	+	+	-
**D3**	Poor	T2N0M0	+	++	-
**D4**	Poor	T2N1M0	++	+++	-
**D5**	moderate/poor	T2N1M0	-	-	-
**D6**	moderate	T2N3M1	-	-	-
**D7**	moderate	T3N3M0	+	+	-
**D8**	moderate	T2N0M0	-	-	-
**D9**	Poor	T2N0M0	-	-	-
**D10**	Poor	T2N0M0	-	-	-
**D11**	Poor	T2N0M0	-	-	-
**E1**	moderate	T2N0M0	-	-	-
**E2**	moderate	T2N0M0	-	-	-
**E3**	moderate	T2N0M0	-	+	-
**E4**	moderate	T2N0MO	-	+++	-
**E5**	moderate	T2N0MO	-	-	-
**E6**	moderate	T2N2M1	+	+	-
**E7**	moderate	T2N0M0	-	-	+++
**E8**	moderate	T2N0M0	-	-	+++

**E9**	moderate	T2N0M0	-	-	+++
**E10**	moderate	T2N0M0	-	-	+++
**E11**	moderate	T2N0M0	-	+	+++
**F1**	moderate	T2N3M0	-	-	-
**F2**	Poor	T2N1M0	-	-	+
**F3**	moderate	T2N0M0	-	-	+++
**F4**	moderate	T2N0M0	-	-	+++
**F5**	moderate	T2N0M0	-	-	-
**F6**	moderate	T2N1M0	+	+	-
**F7**	moderate	T2N0M0	-	-	-
**F8**	moderate	T2N0M0	-	-	-
**F9**	moderate	T2N0M0	-	-	-
**F10**	moderate	TxNxMx	-	-	-
**F11**	moderate/poor	TxNxMx	+	+	-

TNM staging

T1: tumour is 2 centimetres (cm) across or less.
T2: tumour is more than 2 cm but no more than 5 cm across.
T3: tumour is bigger than 5 cm across.
TX: the tumour size can't be assessed.

N0: No cancer was found in the lymph nodes or only areas of cancer smaller than 0.2 mm are in the lymph nodes.
N1: cancer has spread to 1 to 3 axillary lymph nodes and/or the internal mammary lymph nodes.
N2: cancer has spread to 4 to 9 axillary lymph nodes. Or, it has spread to the internal mammary lymph nodes, but not the axillary lymph nodes.
N3: cancer has spread to 10 or more axillary lymph nodes, or it has spread to the lymph nodes located under the clavicle, or collarbone.

M0: there is no sign that the cancer has spread (No distal metastases).
M1: cancer has spread to another part of the body (Distal metastases).

Supplementary Table 2. FACS analysis of Notch1 and Notch2 expression in BrCa cell lines

Cell Line	Subtype	Notch1 positive cells (%)	Notch2 positive cells (%)
MDA-MB231	Basal	2.10	2.58
ZR75D	Luminal B	2.33	1.55
4T1	-	3.14	1.72
BT474	Luminal B	0.00	3.26
T47D	Luminal A	2.81	0.06
MCF-7	Luminal A	0.00	0.00

Supplementary Table 3. Statistically significant GO terms associated with upregulated and downregulated transcripts found in Notch1^{HIGH} vs Notch1^{LOW}

ID	Ontology Term	Description	p.adjust
		**Up-regulated mRNAs**	
GO:0030374	MF	nuclear receptor transcription coactivator activity	0.001179
GO:0003713	MF	transcription coactivator activity	0.014041
GO:0042974	MF	retinoic acid receptor binding	0.02894
GO:0035257	MF	nuclear hormone receptor binding	0.02894
GO:0001085	MF	RNA polymerase II transcription factor binding	0.039815
GO:0003712	MF	transcription coregulator activity	0.04891
GO:0062023	CC	collagen-containing extracellular matrix	0.030611
GO:0005604	CC	basement membrane	0.030611
GO:0005788	CC	endoplasmic reticulum lumen	0.030611
		**Down-regulated mRNAs**	
GO:0016570	BP	histone modification	1.7132E-06
GO:0016569	BP	covalent chromatin modification	1.7132E-06
GO:0018205	BP	peptidyl-lysine modification	1.1157E-05
GO:1904837	BP	beta-catenin-TCF complex assembly	0.00024824
GO:0060541	BP	respiratory system development	0.00035603
GO:0060560	BP	developmental growth involved in morphogenesis	0.00137246
GO:0022604	BP	regulation of cell morphogenesis	0.00245783
GO:0003401	BP	axis elongation	0.0031953
GO:0035107	BP	appendage morphogenesis	0.0031953
GO:0035108	BP	limb morphogenesis	0.0031953
GO:0006096	BP	glycolytic process	0.0031953
GO:0006757	BP	ATP generation from ADP	0.0031953
GO:0030518	BP	intracellular steroid hormone receptor signaling pathway	0.0031953
GO:0050769	BP	positive regulation of neurogenesis	0.0031953
GO:0035855	BP	megakaryocyte development	0.0031953
GO:0060562	BP	epithelial tube morphogenesis	0.0031953
GO:0002011	BP	morphogenesis of an epithelial sheet	0.0031953
GO:0046031	BP	ADP metabolic process	0.00405661
GO:0060602	BP	branch elongation of an epithelium	0.00411704
GO:0018393	BP	internal peptidyl-lysine acetylation	0.00411704
GO:0006475	BP	internal protein amino acid acetylation	0.00431456
GO:0018394	BP	peptidyl-lysine acetylation	0.00508878
GO:0006165	BP	nucleoside diphosphate phosphorylation	0.00508878
GO:0046939	BP	nucleotide phosphorylation	0.00508878
GO:0009135	BP	purine nucleoside diphosphate metabolic process	0.00508878
GO:0009179	BP	purine ribonucleoside diphosphate metabolic process	0.00508878
GO:0030522	BP	intracellular receptor signaling pathway	0.00508878
GO:0006333	BP	chromatin assembly or disassembly	0.00508878
GO:0030324	BP	lung development	0.00508878
GO:0030521	BP	androgen receptor signaling pathway	0.00508878
GO:0009185	BP	ribonucleoside diphosphate metabolic process	0.00508878
GO:0043401	BP	steroid hormone mediated signaling pathway	0.00508878
GO:0030323	BP	respiratory tube development	0.00508878
GO:0048736	BP	appendage development	0.00508878
GO:0060173	BP	limb development	0.00508878
GO:0016571	BP	histone methylation	0.00562016

GO:0034329	BP	cell junction assembly	0.00576512
GO:0033143	BP	regulation of intracellular steroid hormone receptor signaling pathway	0.00622482
GO:0007411	BP	axon guidance	0.00663036
GO:0048732	BP	gland development	0.00663036
GO:0097485	BP	neuron projection guidance	0.0066623
GO:0006090	BP	pyruvate metabolic process	0.00733393
GO:0019058	BP	viral life cycle	0.00733393
GO:0045787	BP	positive regulation of cell cycle	0.00741869
GO:0009132	BP	nucleoside diphosphate metabolic process	0.00787742
GO:0060964	BP	regulation of gene silencing by miRNA	0.00787742
GO:0042789	BP	mRNA transcription by RNA polymerase II	0.00834277
GO:0043543	BP	protein acylation	0.00834277
GO:0006352	BP	DNA-templated transcription, initiation	0.00839341
GO:0016573	BP	histone acetylation	0.00839341
GO:0006473	BP	protein acetylation	0.00839341
GO:0060147	BP	regulation of posttranscriptional gene silencing	0.00845034
GO:0060966	BP	regulation of gene silencing by RNA	0.00845034
GO:0071383	BP	cellular response to steroid hormone stimulus	0.00885304
GO:0022612	BP	gland morphogenesis	0.00998258
GO:0010769	BP	regulation of cell morphogenesis involved in differentiation	0.01019333
GO:0001667	BP	ameboidal-type cell migration	0.01063555
GO:0030326	BP	embryonic limb morphogenesis	0.01063555
GO:0035113	BP	embryonic appendage morphogenesis	0.01063555
GO:0007409	BP	axonogenesis	0.01063555
GO:0045665	BP	negative regulation of neuron differentiation	0.01510151
GO:0006476	BP	protein deacetylation	0.01558847
GO:0098751	BP	bone cell development	0.01558847
GO:0034728	BP	nucleosome organization	0.01697126
GO:0006479	BP	protein methylation	0.01697126
GO:0008213	BP	protein alkylation	0.01697126
GO:0030219	BP	megakaryocyte differentiation	0.01697126
GO:0060968	BP	regulation of gene silencing	0.01697126
GO:0050770	BP	regulation of axonogenesis	0.01758502
GO:0009896	BP	positive regulation of catabolic process	0.01758502
GO:1901201	BP	regulation of extracellular matrix assembly	0.0179656
GO:2001224	BP	positive regulation of neuron migration	0.0179656
GO:0010721	BP	negative regulation of cell development	0.01850563
GO:0009299	BP	mRNA transcription	0.02078561
GO:2000136	BP	regulation of cell proliferation involved in heart morphogenesis	0.02078561
GO:0050768	BP	negative regulation of neurogenesis	0.02078561
GO:0043967	BP	histone H4 acetylation	0.02078561
GO:0071526	BP	semaphorin-plexin signaling pathway	0.02078561
GO:0035601	BP	protein deacylation	0.02078561
GO:0007219	BP	Notch signaling pathway	0.02078561
GO:0031497	BP	chromatin assembly	0.02078561
GO:0008593	BP	regulation of Notch signaling pathway	0.02103911
GO:0035148	BP	tube formation	0.02103911
GO:0003002	BP	regionalization	0.02141038

GO:0007221	BP	positive regulation of transcription of Notch receptor target	0.02161295
GO:0061323	BP	cell proliferation involved in heart morphogenesis	0.02161295
GO:0048562	BP	embryonic organ morphogenesis	0.0220229
GO:0051145	BP	smooth muscle cell differentiation	0.02207073
GO:0009755	BP	hormone-mediated signaling pathway	0.02207073
GO:0016052	BP	carbohydrate catabolic process	0.02207073
GO:0098732	BP	macromolecule deacylation	0.02207073
GO:0048754	BP	branching morphogenesis of an epithelial tube	0.02306034
GO:0045773	BP	positive regulation of axon extension	0.02344415
GO:0008360	BP	regulation of cell shape	0.02344415
GO:0060348	BP	bone development	0.02381792
GO:0010770	BP	positive regulation of cell morphogenesis involved in differentiation	0.02383802
GO:0085029	BP	extracellular matrix assembly	0.02456292
GO:0003007	BP	heart morphogenesis	0.025221
GO:0042771	BP	intrinsic apoptotic signaling pathway in response to DNA damage by p53 class mediator	0.02590365
GO:2001222	BP	regulation of neuron migration	0.02590365
GO:0030099	BP	myeloid cell differentiation	0.02590365
GO:0072332	BP	intrinsic apoptotic signaling pathway by p53 class mediator	0.02590365
GO:0045216	BP	cell-cell junction organization	0.02651294
GO:1900034	BP	regulation of cellular response to heat	0.02691649
GO:0051961	BP	negative regulation of nervous system development	0.02697091
GO:0035050	BP	embryonic heart tube development	0.02697091
GO:1901224	BP	positive regulation of NIK/NF-kappaB signaling	0.02697091
GO:0035357	BP	peroxisome proliferator activated receptor signaling pathway	0.02697091
GO:0080182	BP	histone H3-K4 trimethylation	0.02697091
GO:1903599	BP	positive regulation of autophagy of mitochondrion	0.02697091
GO:0016925	BP	protein sumoylation	0.02984293
GO:0060538	BP	skeletal muscle organ development	0.02985684
GO:0001654	BP	eye development	0.02985684
GO:0002089	BP	lens morphogenesis in camera-type eye	0.02985684
GO:0048675	BP	axon extension	0.02985684
GO:0006338	BP	chromatin remodeling	0.02993697
GO:0009952	BP	anterior/posterior pattern specification	0.03056189
GO:0150063	BP	visual system development	0.03109689
GO:0001838	BP	embryonic epithelial tube formation	0.03109689
GO:0034605	BP	cellular response to heat	0.03109689
GO:0006417	BP	regulation of translation	0.03118222
GO:0048568	BP	embryonic organ development	0.03118222
GO:0031331	BP	positive regulation of cellular catabolic process	0.03118222
GO:0034063	BP	stress granule assembly	0.03118222
GO:0032388	BP	positive regulation of intracellular transport	0.03118222
GO:0043010	BP	camera-type eye development	0.03118222
GO:0098742	BP	cell-cell adhesion via plasma-membrane adhesion molecules	0.03197806
GO:0050772	BP	positive regulation of axonogenesis	0.03204265
GO:0007389	BP	pattern specification process	0.03221364
GO:0048880	BP	sensory system development	0.03221364

GO:0048745	BP	smooth muscle tissue development	0.03349652
GO:2000637	BP	positive regulation of gene silencing by miRNA	0.03349652
GO:0060425	BP	lung morphogenesis	0.03368352
GO:0071824	BP	protein-DNA complex subunit organization	0.03428147
GO:0010508	BP	positive regulation of autophagy	0.03511047
GO:0061053	BP	somite development	0.03511047
GO:0018023	BP	peptidyl-lysine trimethylation	0.03511047
GO:0060148	BP	positive regulation of posttranscriptional gene silencing	0.03615681
GO:0072175	BP	epithelial tube formation	0.03688049
GO:0008361	BP	regulation of cell size	0.03729021
GO:0048545	BP	response to steroid hormone	0.03782203
GO:0007517	BP	muscle organ development	0.03809083
GO:0010506	BP	regulation of autophagy	0.03809083
GO:0022029	BP	telencephalon cell migration	0.03810456
GO:0045444	BP	fat cell differentiation	0.03810456
GO:0060740	BP	prostate gland epithelium morphogenesis	0.03810456
GO:0060765	BP	regulation of androgen receptor signaling pathway	0.03810456
GO:0048588	BP	developmental cell growth	0.03977418
GO:0006735	BP	NADH regeneration	0.04065072
GO:0061621	BP	canonical glycolysis	0.04065072
GO:0061718	BP	glucose catabolic process to pyruvate	0.04065072
GO:0061138	BP	morphogenesis of a branching epithelium	0.04065072
GO:0030520	BP	intracellular estrogen receptor signaling pathway	0.04065072
GO:0045747	BP	positive regulation of Notch signaling pathway	0.04065072
GO:0051568	BP	histone H3-K4 methylation	0.04065072
GO:0006323	BP	DNA packaging	0.04080679
GO:0006367	BP	transcription initiation from RNA polymerase II promoter	0.04247672
GO:0021885	BP	forebrain cell migration	0.0425197
GO:0060512	BP	prostate gland morphogenesis	0.04309819
GO:0061620	BP	glycolytic process through glucose-6-phosphate	0.04309819
GO:0030516	BP	regulation of axon extension	0.04309819
GO:1901655	BP	cellular response to ketone	0.04309819
GO:0030879	BP	mammary gland development	0.04376648
GO:0090068	BP	positive regulation of cell cycle process	0.04458657
GO:0051817	BP	modulation of process of other organism involved in symbiotic interaction	0.04561941
GO:0016049	BP	cell growth	0.04561941
GO:0035116	BP	embryonic hindlimb morphogenesis	0.04561941
GO:0060441	BP	epithelial tube branching involved in lung morphogenesis	0.04561941
GO:0061615	BP	glycolytic process through fructose-6-phosphate	0.04561941
GO:0019080	BP	viral gene expression	0.04662648
GO:0035282	BP	segmentation	0.04662648
GO:0042063	BP	gliogenesis	0.04726137
GO:0051098	BP	regulation of binding	0.04726137
GO:0030511	BP	positive regulation of transforming growth factor beta receptor signaling pathway	0.0483355
GO:0090162	BP	establishment of epithelial cell polarity	0.0483355
GO:1903846	BP	positive regulation of cellular response to transforming growth factor beta stimulus	0.0483355

GO:0050808	BP	synapse organization	0.04932563
GO:0046034	BP	ATP metabolic process	0.04995911
GO:0005667	CC	transcription regulator complex	0.00691938
GO:0010494	CC	cytoplasmic stress granule	0.00691938
GO:0035770	CC	ribonucleoprotein granule	0.00691938
GO:0000123	CC	histone acetyltransferase complex	0.00691938
GO:0000812	CC	Swr1 complex	0.00691938
GO:0048188	CC	Set1C/COMPASS complex	0.00691938
GO:0071006	CC	U2-type catalytic step 1 spliceosome	0.00691938
GO:0071012	CC	catalytic step 1 spliceosome	0.00691938
GO:0031248	CC	protein acetyltransferase complex	0.00808508
GO:1902493	CC	acetyltransferase complex	0.00808508
GO:0036464	CC	cytoplasmic ribonucleoprotein granule	0.01107767
GO:0005844	CC	polysome	0.01312954
GO:0005925	CC	focal adhesion	0.01467833
GO:0070603	CC	SWI/SNF superfamily-type complex	0.0150388
GO:0030055	CC	cell-substrate junction	0.0150388
GO:0035097	CC	histone methyltransferase complex	0.01536085
GO:0005911	CC	cell-cell junction	0.01536085
GO:1904949	CC	ATPase complex	0.01536085
GO:0016528	CC	sarcoplasm	0.016323
GO:0097346	CC	INO80-type complex	0.02644599
GO:0070461	CC	SAGA-type complex	0.03514001
GO:0034708	CC	methyltransferase complex	0.04690908
GO:0003713	MF	transcription coactivator activity	4.1044E-09
GO:0003712	MF	transcription coregulator activity	4.1044E-09
GO:0030374	MF	nuclear receptor transcription coactivator activity	6.8153E-06
GO:0140297	MF	DNA-binding transcription factor binding	3.2863E-05
GO:0035257	MF	nuclear hormone receptor binding	0.00012486
GO:0061629	MF	RNA polymerase II-specific DNA-binding transcription factor binding	0.00015907
GO:0031490	MF	chromatin DNA binding	0.00049434
GO:0051427	MF	hormone receptor binding	0.00049434
GO:0001085	MF	RNA polymerase II transcription factor binding	0.00077508
GO:0140030	MF	modification-dependent protein binding	0.00080426
GO:0070577	MF	lysine-acetylated histone binding	0.00186457
GO:0140033	MF	acetylation-dependent protein binding	0.00186457
GO:0016922	MF	nuclear receptor binding	0.00186457
GO:0042393	MF	histone binding	0.0034586
GO:0042974	MF	retinoic acid receptor binding	0.0034586
GO:0097157	MF	pre-mRNA intronic binding	0.00471015
GO:0045296	MF	cadherin binding	0.01035289
GO:0002039	MF	p53 binding	0.01035289
GO:0042800	MF	histone methyltransferase activity (H3-K4 specific)	0.01182492
GO:0046332	MF	SMAD binding	0.02121103
GO:0035258	MF	steroid hormone receptor binding	0.02260704
GO:0003779	MF	actin binding	0.02325086
GO:0008013	MF	beta-catenin binding	0.0256079
GO:0004402	MF	histone acetyltransferase activity	0.03393447
GO:0008139	MF	nuclear localization sequence binding	0.03573326
GO:0061733	MF	peptide-lysine-N-acetyltransferase activity	0.03573326
GO:0017056	MF	structural constituent of nuclear pore	0.0363568

GO:0008022	MF	protein C-terminus binding	0.0363568
GO:0042054	MF	histone methyltransferase activity	0.0363568
GO:0003714	MF	transcription corepressor activity	0.03940797
GO:0046966	MF	thyroid hormone receptor binding	0.03940797
GO:0016407	MF	acetyltransferase activity	0.04408137
GO:0034212	MF	peptide N-acetyltransferase activity	0.04408137
GO:0036002	MF	pre-mRNA binding	0.04408137
GO:0008190	MF	eukaryotic initiation factor 4E binding	0.04601966

BP: Biological Process; CC: Cellular Component; MF: Molecular Function

Supplementary Table 4. Statistically significant GO terms associated with upregulated and downregulated transcripts found in Notch2^{HIGH} vs Notch2^{LOW}

ID	GO Term	Description	p.adjust
		**Up-regulated mRNAs**	
GO:0030198	BP	extracellular matrix organization	5.4289E-09
GO:0043062	BP	extracellular structure organization	5.4289E-09
GO:0051924	BP	regulation of calcium ion transport	1.0287E-06
GO:0034612	BP	response to tumor necrosis factor	1.3635E-05
GO:0071356	BP	cellular response to tumor necrosis factor	1.4469E-05
GO:0043122	BP	regulation of I-kappaB kinase/NF-kappaB signaling	1.8844E-05
GO:0060337	BP	type I interferon signaling pathway	1.8844E-05
GO:0071357	BP	cellular response to type I interferon	1.8844E-05
GO:0007249	BP	I-kappaB kinase/NF-kappaB signaling	2.7672E-05
GO:0034340	BP	response to type I interferon	2.7672E-05
GO:1903169	BP	regulation of calcium ion transmembrane transport	2.7672E-05
GO:0043123	BP	positive regulation of I-kappaB kinase/NF-kappaB signaling	2.7916E-05
GO:0009636	BP	response to toxic substance	2.7916E-05
GO:0006816	BP	calcium ion transport	6.328E-05
GO:0010959	BP	regulation of metal ion transport	0.00014582
GO:0070838	BP	divalent metal ion transport	0.00014737
GO:0072511	BP	divalent inorganic cation transport	0.0002104
GO:1904062	BP	regulation of cation transmembrane transport	0.00066724
GO:0007204	BP	positive regulation of cytosolic calcium ion concentration	0.00071632
GO:0006979	BP	response to oxidative stress	0.00183093
GO:0033209	BP	tumor necrosis factor-mediated signaling pathway	0.00208928
GO:0010522	BP	regulation of calcium ion transport into cytosol	0.00209523
GO:0051279	BP	regulation of release of sequestered calcium ion into cytosol	0.0025461
GO:0062197	BP	cellular response to chemical stress	0.0025461
GO:0006874	BP	cellular calcium ion homeostasis	0.00300748
GO:0070482	BP	response to oxygen levels	0.00324968
GO:0045785	BP	positive regulation of cell adhesion	0.00331179
GO:0051235	BP	maintenance of location	0.0037373
GO:0036293	BP	response to decreased oxygen levels	0.00400206
GO:0051480	BP	regulation of cytosolic calcium ion concentration	0.00402899
GO:0070588	BP	calcium ion transmembrane transport	0.00402899
GO:0055074	BP	calcium ion homeostasis	0.00421127
GO:0051928	BP	positive regulation of calcium ion transport	0.00421127
GO:0034765	BP	regulation of ion transmembrane transport	0.00515785
GO:0032963	BP	collagen metabolic process	0.00556272
GO:0072503	BP	cellular divalent inorganic cation homeostasis	0.00561134
GO:0048015	BP	phosphatidylinositol-mediated signaling	0.00612712
GO:0014065	BP	phosphatidylinositol 3-kinase signaling	0.00711481
GO:0072659	BP	protein localization to plasma membrane	0.00722736
GO:0061098	BP	positive regulation of protein tyrosine kinase activity	0.00722736
GO:0061097	BP	regulation of protein tyrosine kinase activity	0.00722736
GO:0048017	BP	inositol lipid-mediated signaling	0.00737821
GO:0001666	BP	response to hypoxia	0.00737821
GO:0050804	BP	modulation of chemical synaptic transmission	0.00737821
GO:0099177	BP	regulation of trans-synaptic signaling	0.00737821

GO:0043270	BP	positive regulation of ion transport	0.00737821
GO:0050921	BP	positive regulation of chemotaxis	0.00737821
GO:1904427	BP	positive regulation of calcium ion transmembrane transport	0.00737821
GO:0002685	BP	regulation of leukocyte migration	0.00737821
GO:0060402	BP	calcium ion transport into cytosol	0.00768673
GO:0010803	BP	regulation of tumor necrosis factor-mediated signaling pathway	0.00781899
GO:0050920	BP	regulation of chemotaxis	0.00845511
GO:0032496	BP	response to lipopolysaccharide	0.0085708
GO:0051651	BP	maintenance of location in cell	0.00891782
GO:0097237	BP	cellular response to toxic substance	0.00907371
GO:0052547	BP	regulation of peptidase activity	0.01027965
GO:0002237	BP	response to molecule of bacterial origin	0.01121043
GO:0060401	BP	cytosolic calcium ion transport	0.01121043
GO:0034599	BP	cellular response to oxidative stress	0.01121043
GO:0043312	BP	neutrophil degranulation	0.01174266
GO:0031589	BP	cell-substrate adhesion	0.01227597
GO:0002283	BP	neutrophil activation involved in immune response	0.01272835
GO:0097553	BP	calcium ion transmembrane import into cytosol	0.01272835
GO:0009313	BP	oligosaccharide catabolic process	0.01283499
GO:0018212	BP	peptidyl-tyrosine modification	0.01283499
GO:0002819	BP	regulation of adaptive immune response	0.01311248
GO:0090200	BP	positive regulation of release of cytochrome c from mitochondria	0.01311248
GO:0070555	BP	response to interleukin-1	0.01352818
GO:0001959	BP	regulation of cytokine-mediated signaling pathway	0.01362621
GO:0002687	BP	positive regulation of leukocyte migration	0.01362621
GO:0033628	BP	regulation of cell adhesion mediated by integrin	0.01609672
GO:0051209	BP	release of sequestered calcium ion into cytosol	0.01609672
GO:0097191	BP	extrinsic apoptotic signaling pathway	0.01683584
GO:0050777	BP	negative regulation of immune response	0.01697205
GO:0051283	BP	negative regulation of sequestering of calcium ion	0.01697205
GO:0022617	BP	extracellular matrix disassembly	0.01706101
GO:0030595	BP	leukocyte chemotaxis	0.01787843
GO:0033627	BP	cell adhesion mediated by integrin	0.01787843
GO:1990778	BP	protein localization to cell periphery	0.01787843
GO:0044409	BP	entry into host	0.01787843
GO:1904064	BP	positive regulation of cation transmembrane transport	0.01787843
GO:0046718	BP	viral entry into host cell	0.01834943
GO:0051282	BP	regulation of sequestering of calcium ion	0.01843425
GO:0018108	BP	peptidyl-tyrosine phosphorylation	0.01864664
GO:0006027	BP	glycosaminoglycan catabolic process	0.01935145
GO:0000302	BP	response to reactive oxygen species	0.01951072
GO:0050727	BP	regulation of inflammatory response	0.01977661
GO:0045824	BP	negative regulation of innate immune response	0.02141487
GO:0043687	BP	post-translational protein modification	0.02196857
GO:0002688	BP	regulation of leukocyte chemotaxis	0.02196857
GO:0051208	BP	sequestering of calcium ion	0.02196857
GO:0002683	BP	negative regulation of immune system process	0.02287489
GO:0009615	BP	response to virus	0.02374148
GO:0048246	BP	macrophage chemotaxis	0.02425745

GO:0022604	BP	regulation of cell morphogenesis	0.02426106
GO:0060759	BP	regulation of response to cytokine stimulus	0.02453178
GO:0014066	BP	regulation of phosphatidylinositol 3-kinase signaling	0.02612992
GO:0002407	BP	dendritic cell chemotaxis	0.02612992
GO:0071347	BP	cellular response to interleukin-1	0.02612992
GO:0046640	BP	regulation of alpha-beta T cell proliferation	0.02612992
GO:1990748	BP	cellular detoxification	0.02623401
GO:0150077	BP	regulation of neuroinflammatory response	0.02641167
GO:0010524	BP	positive regulation of calcium ion transport into cytosol	0.02782976
GO:0031667	BP	response to nutrient levels	0.0300298
GO:0002228	BP	natural killer cell mediated immunity	0.0300298
GO:1904019	BP	epithelial cell apoptotic process	0.03254108
GO:0050730	BP	regulation of peptidyl-tyrosine phosphorylation	0.03256899
GO:0006026	BP	aminoglycan catabolic process	0.03292591
GO:0002831	BP	regulation of response to biotic stimulus	0.03292591
GO:0033559	BP	unsaturated fatty acid metabolic process	0.03399704
GO:0044273	BP	sulfur compound catabolic process	0.03399704
GO:0031664	BP	regulation of lipopolysaccharide-mediated signaling pathway	0.03663466
GO:0034767	BP	positive regulation of ion transmembrane transport	0.0368373
GO:0019233	BP	sensory perception of pain	0.0368373
GO:1905517	BP	macrophage migration	0.03746059
GO:0046633	BP	alpha-beta T cell proliferation	0.03921228
GO:0016485	BP	protein processing	0.04176004
GO:0030574	BP	collagen catabolic process	0.04176004
GO:0032480	BP	negative regulation of type I interferon production	0.04176004
GO:0052548	BP	regulation of endopeptidase activity	0.04176004
GO:0033632	BP	regulation of cell-cell adhesion mediated by integrin	0.04176004
GO:0097530	BP	granulocyte migration	0.04313181
GO:0031663	BP	lipopolysaccharide-mediated signaling pathway	0.04521247
GO:0061041	BP	regulation of wound healing	0.04525268
GO:0042982	BP	amyloid precursor protein metabolic process	0.04525268
GO:0022407	BP	regulation of cell-cell adhesion	0.04833411
GO:0098754	BP	detoxification	0.04852399
GO:0046596	BP	regulation of viral entry into host cell	0.04974815
GO:0005788	CC	endoplasmic reticulum lumen	1.7974E-07
GO:0062023	CC	collagen-containing extracellular matrix	3.1806E-07
GO:0043202	CC	lysosomal lumen	6.4342E-06
GO:0005775	CC	vacuolar lumen	6.5985E-06
GO:0005604	CC	basement membrane	0.00029052
GO:0034774	CC	secretory granule lumen	0.00065417
GO:0060205	CC	cytoplasmic vesicle lumen	0.00072275
GO:0031983	CC	vesicle lumen	0.00072275
GO:0005911	CC	cell-cell junction	0.00117786
GO:0098644	CC	complex of collagen trimers	0.00117786
GO:0005774	CC	vacuolar membrane	0.00190393
GO:0035578	CC	azurophil granule lumen	0.00203453
GO:0030133	CC	transport vesicle	0.00335608
GO:0030017	CC	sarcomere	0.00520635
GO:0043292	CC	contractile fiber	0.00520635
GO:0005766	CC	primary lysosome	0.00520635
GO:0042582	CC	azurophil granule	0.00520635

GO:0001725	CC	stress fiber	0.00520635
GO:0097517	CC	contractile actin filament bundle	0.00520635
GO:0030016	CC	myofibril	0.00540253
GO:0042581	CC	specific granule	0.0069054
GO:0005765	CC	lysosomal membrane	0.00768235
GO:0098852	CC	lytic vacuole membrane	0.00768235
GO:0031674	CC	I band	0.01070824
GO:0032432	CC	actin filament bundle	0.01070824
GO:0042641	CC	actomyosin	0.01440052
GO:0031300	CC	intrinsic component of organelle membrane	0.01808721
GO:0031252	CC	cell leading edge	0.02242901
GO:0005583	CC	fibrillar collagen trimer	0.02517467
GO:0098643	CC	banded collagen fibril	0.02517467
GO:0005581	CC	collagen trimer	0.02715243
GO:0030018	CC	Z disc	0.02735778
GO:0070382	CC	exocytic vesicle	0.03698368
GO:1904724	CC	tertiary granule lumen	0.04334867
GO:0005912	CC	adherens junction	0.04517864
GO:0016324	CC	apical plasma membrane	0.04593356
GO:0005201	MF	extracellular matrix structural constituent	6.6839E-05
GO:0030020	MF	extracellular matrix structural constituent conferring tensile strength	0.03396013
GO:0005178	MF	integrin binding	0.03396013
GO:0030296	MF	protein tyrosine kinase activator activity	0.03396013
**Down-regulated mRNAs**			
GO:0016569	BP	covalent chromatin modification	5.2763E-14
GO:0016570	BP	histone modification	5.6491E-13
GO:0018205	BP	peptidyl-lysine modification	1.0602E-12
GO:0033044	BP	regulation of chromosome organization	4.3152E-08
GO:0008380	BP	RNA splicing	4.2616E-07
GO:0045787	BP	positive regulation of cell cycle	7.1436E-07
GO:0006338	BP	chromatin remodeling	1.0991E-06
GO:0016571	BP	histone methylation	1.8405E-06
GO:0090068	BP	positive regulation of cell cycle process	2.0376E-06
GO:0006260	BP	DNA replication	2.1686E-06
GO:0000377	BP	RNA splicing, via transesterification reactions with bulged adenosine as nucleophile	2.5565E-06
GO:0000398	BP	mRNA splicing, via spliceosome	2.5565E-06
GO:0000375	BP	RNA splicing, via transesterification reactions	2.9096E-06
GO:0034968	BP	histone lysine methylation	4.3047E-06
GO:2001252	BP	positive regulation of chromosome organization	4.4174E-06
GO:0006611	BP	protein export from nucleus	5.1407E-06
GO:0051169	BP	nuclear transport	6.1141E-06
GO:0051236	BP	establishment of RNA localization	7.3893E-06
GO:0043923	BP	positive regulation by host of viral transcription	7.3893E-06
GO:0051168	BP	nuclear export	7.3893E-06
GO:0006403	BP	RNA localization	8.3208E-06
GO:0072331	BP	signal transduction by p53 class mediator	8.3208E-06
GO:0018393	BP	internal peptidyl-lysine acetylation	8.3675E-06
GO:0051568	BP	histone H3-K4 methylation	8.5069E-06
GO:0006406	BP	mRNA export from nucleus	8.6867E-06

GO:0071427	BP	mRNA-containing ribonucleoprotein complex export from nucleus	8.6867E-06
GO:0006475	BP	internal protein amino acid acetylation	8.893E-06
GO:1902275	BP	regulation of chromatin organization	9.1216E-06
GO:0006913	BP	nucleocytoplasmic transport	9.4038E-06
GO:0071103	BP	DNA conformation change	9.7349E-06
GO:0071426	BP	ribonucleoprotein complex export from nucleus	1.2933E-05
GO:0006405	BP	RNA export from nucleus	1.3422E-05
GO:0071166	BP	ribonucleoprotein complex localization	1.3422E-05
GO:0018394	BP	peptidyl-lysine acetylation	1.3422E-05
GO:0016573	BP	histone acetylation	1.4276E-05
GO:0018022	BP	peptidyl-lysine methylation	1.4276E-05
GO:0006261	BP	DNA-dependent DNA replication	2.6236E-05
GO:0051028	BP	mRNA transport	2.8492E-05
GO:0006479	BP	protein methylation	3.7694E-05
GO:0008213	BP	protein alkylation	3.7694E-05
GO:0006302	BP	double-strand break repair	3.7906E-05
GO:0050657	BP	nucleic acid transport	3.8559E-05
GO:0050658	BP	RNA transport	3.8559E-05
GO:0071824	BP	protein-DNA complex subunit organization	4.6186E-05
GO:0006473	BP	protein acetylation	4.8102E-05
GO:0006333	BP	chromatin assembly or disassembly	5.3632E-05
GO:0070507	BP	regulation of microtubule cytoskeleton organization	5.5994E-05
GO:0031570	BP	DNA integrity checkpoint	5.7719E-05
GO:0031062	BP	positive regulation of histone methylation	7.6691E-05
GO:0031503	BP	protein-containing complex localization	7.6691E-05
GO:0006352	BP	DNA-templated transcription, initiation	0.00013308
GO:0034401	BP	chromatin organization involved in regulation of transcription	0.00013681
GO:0044786	BP	cell cycle DNA replication	0.00014351
GO:0000075	BP	cell cycle checkpoint	0.00015172
GO:1901990	BP	regulation of mitotic cell cycle phase transition	0.00015598
GO:1901987	BP	regulation of cell cycle phase transition	0.00015598
GO:0030518	BP	intracellular steroid hormone receptor signaling pathway	0.00015796
GO:0043044	BP	ATP-dependent chromatin remodeling	0.00015796
GO:0032200	BP	telomere organization	0.00016132
GO:0031060	BP	regulation of histone methylation	0.00017733
GO:0000723	BP	telomere maintenance	0.00018795
GO:1903311	BP	regulation of mRNA metabolic process	0.00019704
GO:1905269	BP	positive regulation of chromatin organization	0.00021431
GO:0007099	BP	centriole replication	0.00021431
GO:1901796	BP	regulation of signal transduction by p53 class mediator	0.00024782
GO:0016925	BP	protein sumoylation	0.00025723
GO:0080182	BP	histone H3-K4 trimethylation	0.00026008
GO:0000077	BP	DNA damage checkpoint	0.00026638
GO:0007059	BP	chromosome segregation	0.00028206
GO:0032259	BP	methylation	0.00028923
GO:0043543	BP	protein acylation	0.00029258
GO:0070646	BP	protein modification by small protein removal	0.00029612
GO:0043414	BP	macromolecule methylation	0.00029782
GO:0043401	BP	steroid hormone mediated signaling pathway	0.00031773
GO:0140014	BP	mitotic nuclear division	0.00035412

GO:0098534	BP	centriole assembly	0.00036192
GO:0030219	BP	megakaryocyte differentiation	0.00042479
GO:0050434	BP	positive regulation of viral transcription	0.00043823
GO:0007062	BP	sister chromatid cohesion	0.00045149
GO:0033143	BP	regulation of intracellular steroid hormone receptor signaling pathway	0.00045149
GO:0032886	BP	regulation of microtubule-based process	0.00047315
GO:0031109	BP	microtubule polymerization or depolymerization	0.00057016
GO:0015931	BP	nucleobase-containing compound transport	0.00059024
GO:1902115	BP	regulation of organelle assembly	0.00059024
GO:0019083	BP	viral transcription	0.00059024
GO:0019080	BP	viral gene expression	0.00062846
GO:2001022	BP	positive regulation of response to DNA damage stimulus	0.00066255
GO:0000819	BP	sister chromatid segregation	0.00066255
GO:0006409	BP	tRNA export from nucleus	0.00070671
GO:0071431	BP	tRNA-containing ribonucleoprotein complex export from nucleus	0.00070671
GO:0016579	BP	protein deubiquitination	0.00071862
GO:0051298	BP	centrosome duplication	0.00082951
GO:0046607	BP	positive regulation of centrosome cycle	0.00082951
GO:0050684	BP	regulation of mRNA processing	0.00082963
GO:0000082	BP	G1/S transition of mitotic cell cycle	0.00087383
GO:0007064	BP	mitotic sister chromatid cohesion	0.00088457
GO:0051571	BP	positive regulation of histone H3-K4 methylation	0.00090508
GO:0098813	BP	nuclear chromosome segregation	0.00100273
GO:0043967	BP	histone H4 acetylation	0.00103046
GO:0044843	BP	cell cycle G1/S phase transition	0.00109854
GO:0006323	BP	DNA packaging	0.00118198
GO:0051031	BP	tRNA transport	0.00121119
GO:0033260	BP	nuclear DNA replication	0.001371
GO:0040029	BP	regulation of gene expression, epigenetic	0.00142861
GO:0097064	BP	ncRNA export from nucleus	0.00144253
GO:1902117	BP	positive regulation of organelle assembly	0.00144253
GO:0051225	BP	spindle assembly	0.00153834
GO:0031124	BP	mRNA 3'-end processing	0.00158774
GO:0051569	BP	regulation of histone H3-K4 methylation	0.00169062
GO:1903706	BP	regulation of hemopoiesis	0.00191572
GO:0031122	BP	cytoplasmic microtubule organization	0.00196686
GO:0031056	BP	regulation of histone modification	0.00201081
GO:1904837	BP	beta-catenin-TCF complex assembly	0.00206477
GO:0007051	BP	spindle organization	0.00218626
GO:0034728	BP	nucleosome organization	0.00218626
GO:0046785	BP	microtubule polymerization	0.00218626
GO:0009755	BP	hormone-mediated signaling pathway	0.00218626
GO:0001654	BP	eye development	0.00224009
GO:0045931	BP	positive regulation of mitotic cell cycle	0.00237436
GO:0000288	BP	nuclear-transcribed mRNA catabolic process, deadenylation-dependent decay	0.00237687
GO:0046782	BP	regulation of viral transcription	0.00237687
GO:0050686	BP	negative regulation of mRNA processing	0.00239792
GO:0031058	BP	positive regulation of histone modification	0.00240414
GO:0018023	BP	peptidyl-lysine trimethylation	0.00241895

GO:0051983	BP	regulation of chromosome segregation	0.00252112
GO:0150063	BP	visual system development	0.00252112
GO:0000724	BP	double-strand break repair via homologous recombination	0.00262359
GO:0006110	BP	regulation of glycolytic process	0.00283367
GO:0071383	BP	cellular response to steroid hormone stimulus	0.00295206
GO:0006367	BP	transcription initiation from RNA polymerase II promoter	0.00295752
GO:0000725	BP	recombinational repair	0.00297627
GO:0060968	BP	regulation of gene silencing	0.00297627
GO:0046599	BP	regulation of centriole replication	0.00308088
GO:0007098	BP	centrosome cycle	0.00308088
GO:0048880	BP	sensory system development	0.00308088
GO:2001020	BP	regulation of response to DNA damage stimulus	0.00327155
GO:0045652	BP	regulation of megakaryocyte differentiation	0.00330244
GO:1903313	BP	positive regulation of mRNA metabolic process	0.00330244
GO:0010824	BP	regulation of centrosome duplication	0.00330451
GO:0006476	BP	protein deacetylation	0.00354492
GO:0097549	BP	chromatin organization involved in negative regulation of transcription	0.00354492
GO:0048511	BP	rhythmic process	0.00423185
GO:0001701	BP	in utero embryonic development	0.00427886
GO:0051052	BP	regulation of DNA metabolic process	0.00455045
GO:0006096	BP	glycolytic process	0.00455045
GO:0033144	BP	negative regulation of intracellular steroid hormone receptor signaling pathway	0.00455045
GO:0046605	BP	regulation of centrosome cycle	0.00482162
GO:0006757	BP	ATP generation from ADP	0.00486223
GO:0060964	BP	regulation of gene silencing by miRNA	0.00486223
GO:1902749	BP	regulation of cell cycle G2/M phase transition	0.00503494
GO:0000086	BP	G2/M transition of mitotic cell cycle	0.00515222
GO:0010389	BP	regulation of G2/M transition of mitotic cell cycle	0.00515222
GO:0051054	BP	positive regulation of DNA metabolic process	0.00515222
GO:0044839	BP	cell cycle G2/M phase transition	0.00518549
GO:0042770	BP	signal transduction in response to DNA damage	0.00518549
GO:0043966	BP	histone H3 acetylation	0.00518549
GO:0022616	BP	DNA strand elongation	0.00549073
GO:0034329	BP	cell junction assembly	0.00605656
GO:0034504	BP	protein localization to nucleus	0.00620831
GO:0061647	BP	histone H3-K9 modification	0.0062525
GO:0060147	BP	regulation of posttranscriptional gene silencing	0.0062525
GO:0060966	BP	regulation of gene silencing by RNA	0.0062525
GO:0031023	BP	microtubule organizing center organization	0.0062525
GO:0045876	BP	positive regulation of sister chromatid cohesion	0.0063319
GO:0051851	BP	modulation by host of symbiont process	0.0063319
GO:0043470	BP	regulation of carbohydrate catabolic process	0.0063479
GO:0031123	BP	RNA 3'-end processing	0.0063479
GO:0032201	BP	telomere maintenance via semi-conservative replication	0.00640803
GO:0051567	BP	histone H3-K9 methylation	0.00675036
GO:0006337	BP	nucleosome disassembly	0.0068494
GO:1900153	BP	positive regulation of nuclear-transcribed mRNA catabolic process, deadenylation-dependent decay	0.0068494
GO:0065004	BP	protein-DNA complex assembly	0.0068494

GO:0030330	BP	DNA damage response, signal transduction by p53 class mediator	0.00729535
GO:0035601	BP	protein deacylation	0.00729535
GO:0043484	BP	regulation of RNA splicing	0.00748396
GO:0046794	BP	transport of virus	0.0075849
GO:0030522	BP	intracellular receptor signaling pathway	0.0079102
GO:0046031	BP	ADP metabolic process	0.00791375
GO:1900034	BP	regulation of cellular response to heat	0.0084254
GO:0006271	BP	DNA strand elongation involved in DNA replication	0.00859036
GO:0006284	BP	base-excision repair	0.00864865
GO:0007028	BP	cytoplasm organization	0.00864865
GO:0051574	BP	positive regulation of histone H3-K9 methylation	0.00864865
GO:0043547	BP	positive regulation of GTPase activity	0.00864865
GO:0060249	BP	anatomical structure homeostasis	0.00885728
GO:0018210	BP	peptidyl-threonine modification	0.00885728
GO:0000280	BP	nuclear division	0.008888
GO:0032922	BP	circadian regulation of gene expression	0.008888
GO:0044766	BP	multi-organism transport	0.008888
GO:1902579	BP	multi-organism localization	0.008888
GO:0031497	BP	chromatin assembly	0.0089676
GO:0098732	BP	macromolecule deacylation	0.00929529
GO:0010212	BP	response to ionizing radiation	0.00953056
GO:0006303	BP	double-strand break repair via nonhomologous end joining	0.00961616
GO:0018105	BP	peptidyl-serine phosphorylation	0.00977707
GO:0006275	BP	regulation of DNA replication	0.00983787
GO:0046822	BP	regulation of nucleocytoplasmic transport	0.00983787
GO:0031498	BP	chromatin disassembly	0.01002029
GO:0032986	BP	protein-DNA complex disassembly	0.01002029
GO:1900151	BP	regulation of nuclear-transcribed mRNA catabolic process, deadenylation-dependent decay	0.01002029
GO:0048024	BP	regulation of mRNA splicing, via spliceosome	0.01045964
GO:0007623	BP	circadian rhythm	0.01115423
GO:0060213	BP	positive regulation of nuclear-transcribed mRNA poly(A) tail shortening	0.01142134
GO:0044782	BP	cilium organization	0.01220699
GO:0030521	BP	androgen receptor signaling pathway	0.01220699
GO:0043087	BP	regulation of GTPase activity	0.01220699
GO:0007093	BP	mitotic cell cycle checkpoint	0.0122338
GO:0060271	BP	cilium assembly	0.0122338
GO:0033962	BP	P-body assembly	0.0122338
GO:0032392	BP	DNA geometric change	0.01270192
GO:0071826	BP	ribonucleoprotein complex subunit organization	0.01270605
GO:0031110	BP	regulation of microtubule polymerization or depolymerization	0.01286755
GO:0006165	BP	nucleoside diphosphate phosphorylation	0.01301692
GO:0006310	BP	DNA recombination	0.01314499
GO:0018107	BP	peptidyl-threonine phosphorylation	0.01340444
GO:0075733	BP	intracellular transport of virus	0.01342592
GO:0045739	BP	positive regulation of DNA repair	0.01346087
GO:1901532	BP	regulation of hematopoietic progenitor cell differentiation	0.01373505
GO:0007569	BP	cell aging	0.01420446

GO:0060996	BP	dendritic spine development	0.01424931
GO:0046939	BP	nucleotide phosphorylation	0.01442401
GO:0007063	BP	regulation of sister chromatid cohesion	0.01442401
GO:0048025	BP	negative regulation of mRNA splicing, via spliceosome	0.01442401
GO:0048096	BP	chromatin-mediated maintenance of transcription	0.0145481
GO:0042771	BP	intrinsic apoptotic signaling pathway in response to DNA damage by p53 class mediator	0.01482518
GO:0043928	BP	exonucleolytic catabolism of deadenylated mRNA	0.01497368
GO:0009135	BP	purine nucleoside diphosphate metabolic process	0.01501434
GO:0009179	BP	purine ribonucleoside diphosphate metabolic process	0.01501434
GO:0045815	BP	positive regulation of gene expression, epigenetic	0.01596674
GO:0002244	BP	hematopoietic progenitor cell differentiation	0.01596674
GO:0000726	BP	non-recombinational repair	0.01598012
GO:2000142	BP	regulation of DNA-templated transcription, initiation	0.01646586
GO:0006109	BP	regulation of carbohydrate metabolic process	0.01692989
GO:0031112	BP	positive regulation of microtubule polymerization or depolymerization	0.01692989
GO:0006359	BP	regulation of transcription by RNA polymerase III	0.01692989
GO:0018027	BP	peptidyl-lysine dimethylation	0.01692989
GO:0030099	BP	myeloid cell differentiation	0.01703424
GO:0009185	BP	ribonucleoside diphosphate metabolic process	0.01763724
GO:0071156	BP	regulation of cell cycle arrest	0.01808909
GO:0030900	BP	forebrain development	0.01808909
GO:0048285	BP	organelle fission	0.01814558
GO:0006999	BP	nuclear pore organization	0.01826969
GO:0060211	BP	regulation of nuclear-transcribed mRNA poly(A) tail shortening	0.01826969
GO:0048524	BP	positive regulation of viral process	0.01910342
GO:0000291	BP	nuclear-transcribed mRNA catabolic process, exonucleolytic	0.01910342
GO:0051101	BP	regulation of DNA binding	0.01961885
GO:0043010	BP	camera-type eye development	0.01965765
GO:0022618	BP	ribonucleoprotein complex assembly	0.01968552
GO:0048701	BP	embryonic cranial skeleton morphogenesis	0.0198968
GO:0032508	BP	DNA duplex unwinding	0.02005067
GO:0044774	BP	mitotic DNA integrity checkpoint	0.02005067
GO:0018209	BP	peptidyl-serine modification	0.02005067
GO:1903578	BP	regulation of ATP metabolic process	0.02046209
GO:0071108	BP	protein K48-linked deubiquitination	0.0214814
GO:0000070	BP	mitotic sister chromatid segregation	0.02176471
GO:0061008	BP	hepaticobiliary system development	0.02270411
GO:0043584	BP	nose development	0.02274602
GO:0044849	BP	estrous cycle	0.02274602
GO:0060766	BP	negative regulation of androgen receptor signaling pathway	0.02274602
GO:0030326	BP	embryonic limb morphogenesis	0.02276528
GO:0035113	BP	embryonic appendage morphogenesis	0.02276528
GO:0007088	BP	regulation of mitotic nuclear division	0.02383847
GO:0061014	BP	positive regulation of mRNA catabolic process	0.02402188
GO:0016575	BP	histone deacetylation	0.02402188
GO:0097711	BP	ciliary basal body-plasma membrane docking	0.02452427

GO:0043161	BP	proteasome-mediated ubiquitin-dependent protein catabolic process	0.02482587
GO:0070828	BP	heterochromatin organization	0.02596909
GO:0031113	BP	regulation of microtubule polymerization	0.02657977
GO:0033119	BP	negative regulation of RNA splicing	0.02657977
GO:0060765	BP	regulation of androgen receptor signaling pathway	0.02657977
GO:2000144	BP	positive regulation of DNA-templated transcription, initiation	0.02693417
GO:1903320	BP	regulation of protein modification by small protein conjugation or removal	0.0274548
GO:0071158	BP	positive regulation of cell cycle arrest	0.0277261
GO:0046831	BP	regulation of RNA export from nucleus	0.02779682
GO:1902969	BP	mitotic DNA replication	0.02779682
GO:0048545	BP	response to steroid hormone	0.02989405
GO:0006270	BP	DNA replication initiation	0.02989405
GO:0031076	BP	embryonic camera-type eye development	0.02989405
GO:0033146	BP	regulation of intracellular estrogen receptor signaling pathway	0.02989405
GO:0046329	BP	negative regulation of JNK cascade	0.02989405
GO:0006606	BP	protein import into nucleus	0.02989405
GO:0051817	BP	modulation of process of other organism involved in symbiotic interaction	0.03144791
GO:0050808	BP	synapse organization	0.03144791
GO:0043392	BP	negative regulation of DNA binding	0.03152868
GO:0022604	BP	regulation of cell morphogenesis	0.03167256
GO:0071479	BP	cellular response to ionizing radiation	0.03257171
GO:0016358	BP	dendrite development	0.03257171
GO:0006090	BP	pyruvate metabolic process	0.03281075
GO:0033045	BP	regulation of sister chromatid segregation	0.03377026
GO:0048596	BP	embryonic camera-type eye morphogenesis	0.03494854
GO:1902903	BP	regulation of supramolecular fiber organization	0.03527584
GO:0044773	BP	mitotic DNA damage checkpoint	0.03527584
GO:0051495	BP	positive regulation of cytoskeleton organization	0.03602825
GO:0043297	BP	apical junction assembly	0.03602825
GO:0010498	BP	proteasomal protein catabolic process	0.03670807
GO:0046825	BP	regulation of protein export from nucleus	0.03683418
GO:0009132	BP	nucleoside diphosphate metabolic process	0.03778962
GO:0001890	BP	placenta development	0.03982264
GO:0007020	BP	microtubule nucleation	0.03982264
GO:0030217	BP	T cell differentiation	0.0403816
GO:1900542	BP	regulation of purine nucleotide metabolic process	0.04175341
GO:0010634	BP	positive regulation of epithelial cell migration	0.0422714
GO:1903312	BP	negative regulation of mRNA metabolic process	0.04405191
GO:0031507	BP	heterochromatin assembly	0.04405191
GO:0033047	BP	regulation of mitotic sister chromatid segregation	0.04405191
GO:0030520	BP	intracellular estrogen receptor signaling pathway	0.0446361
GO:0031116	BP	positive regulation of microtubule polymerization	0.04525255
GO:0006140	BP	regulation of nucleotide metabolic process	0.04634102
GO:0001889	BP	liver development	0.04634918
GO:0042795	BP	snRNA transcription by RNA polymerase II	0.04694966
GO:0072401	BP	signal transduction involved in DNA integrity checkpoint	0.04694966
GO:0072422	BP	signal transduction involved in DNA damage checkpoint	0.04694966

GO:0032239	BP	regulation of nucleobase-containing compound transport	0.04794408
GO:0007224	BP	smoothened signaling pathway	0.04831814
GO:0051258	BP	protein polymerization	0.0488926
GO:0032465	BP	regulation of cytokinesis	0.04948773
GO:0016607	CC	nuclear speck	8.0823E-15
GO:0005667	CC	transcription regulator complex	1.2463E-09
GO:0098687	CC	chromosomal region	1.5934E-07
GO:0034399	CC	nuclear periphery	3.8661E-07
GO:0017053	CC	transcription repressor complex	3.1647E-06
GO:0005819	CC	spindle	3.2193E-06
GO:0000118	CC	histone deacetylase complex	1.9072E-05
GO:1904949	CC	ATPase complex	1.9713E-05
GO:0016363	CC	nuclear matrix	4.4487E-05
GO:0070603	CC	SWI/SNF superfamily-type complex	5.2382E-05
GO:0071564	CC	npBAF complex	5.2382E-05
GO:0005814	CC	centriole	6.7645E-05
GO:0016514	CC	SWI/SNF complex	6.7645E-05
GO:0090575	CC	RNA polymerase II transcription regulator complex	8.331E-05
GO:0071565	CC	nBAF complex	0.0001907
GO:0001650	CC	fibrillar center	0.0002389
GO:0000781	CC	chromosome, telomeric region	0.00034977
GO:0000922	CC	spindle pole	0.00035932
GO:0005635	CC	nuclear envelope	0.00036637
GO:0000793	CC	condensed chromosome	0.00057589
GO:0071013	CC	catalytic step 2 spliceosome	0.00083637
GO:0031965	CC	nuclear membrane	0.00083637
GO:0000775	CC	chromosome, centromeric region	0.000841
GO:0016605	CC	PML body	0.000841
GO:0018995	CC	host cellular component	0.00119394
GO:0043657	CC	host cell	0.00119394
GO:0071011	CC	precatalytic spliceosome	0.0012067
GO:0000123	CC	histone acetyltransferase complex	0.00195487
GO:0035327	CC	transcriptionally active chromatin	0.0020442
GO:0005643	CC	nuclear pore	0.00226876
GO:0032993	CC	protein-DNA complex	0.00355842
GO:0034708	CC	methyltransferase complex	0.00355842
GO:0071005	CC	U2-type precatalytic spliceosome	0.00377782
GO:0031527	CC	filopodium membrane	0.00377782
GO:0035097	CC	histone methyltransferase complex	0.00377782
GO:0005681	CC	spliceosomal complex	0.00401052
GO:0031248	CC	protein acetyltransferase complex	0.00401052
GO:1902493	CC	acetyltransferase complex	0.00401052
GO:0036464	CC	cytoplasmic ribonucleoprotein granule	0.00496625
GO:0043596	CC	nuclear replication fork	0.00513321
GO:0030496	CC	midbody	0.00588922
GO:0000930	CC	gamma-tubulin complex	0.00681802
GO:0005657	CC	replication fork	0.00738582
GO:0035770	CC	ribonucleoprotein granule	0.00738582
GO:0042575	CC	DNA polymerase complex	0.00800753
GO:0005874	CC	microtubule	0.00887952
GO:0005669	CC	transcription factor TFIID complex	0.00945221
GO:0000346	CC	transcription export complex	0.01022976

GO:0008278	CC	cohesin complex	0.01022976
GO:0000784	CC	nuclear chromosome, telomeric region	0.01039584
GO:0005684	CC	U2-type spliceosomal complex	0.0117826
GO:0000792	CC	heterochromatin	0.01218573
GO:0005871	CC	kinesin complex	0.01490053
GO:0070822	CC	Sin3-type complex	0.01599159
GO:0072686	CC	mitotic spindle	0.01730692
GO:0016592	CC	mediator complex	0.02171014
GO:0055029	CC	nuclear DNA-directed RNA polymerase complex	0.02279537
GO:0000428	CC	DNA-directed RNA polymerase complex	0.02405354
GO:0097431	CC	mitotic spindle pole	0.02643679
GO:0090543	CC	Flemming body	0.03009923
GO:0030880	CC	RNA polymerase complex	0.03009923
GO:0005637	CC	nuclear inner membrane	0.0327219
GO:0000242	CC	pericentriolar material	0.03941446
GO:0031010	CC	ISWI-type complex	0.03985658
GO:1990907	CC	beta-catenin-TCF complex	0.03985658
GO:0016591	CC	RNA polymerase II, holoenzyme	0.03985658
GO:0061695	CC	transferase complex, transferring phosphorus-containing groups	0.04386864
GO:0000812	CC	Swr1 complex	0.04610531
GO:0042405	CC	nuclear inclusion body	0.04610531
GO:0042555	CC	MCM complex	0.04610531
GO:0044615	CC	nuclear pore nuclear basket	0.04610531
GO:0048188	CC	Set1C/COMPASS complex	0.04610531
GO:0071006	CC	U2-type catalytic step 1 spliceosome	0.04610531
GO:0071012	CC	catalytic step 1 spliceosome	0.04610531
GO:0000776	CC	kinetochore	0.0466345
GO:0042393	MF	histone binding	1.7609E-14
GO:0003712	MF	transcription coregulator activity	2.2549E-14
GO:0003713	MF	transcription coactivator activity	4.8217E-09
GO:0140297	MF	DNA-binding transcription factor binding	3.7392E-07
GO:0070577	MF	lysine-acetylated histone binding	2.8211E-06
GO:0140033	MF	acetylation-dependent protein binding	2.8211E-06
GO:0016887	MF	ATPase activity	4.5354E-06
GO:0004386	MF	helicase activity	1.8057E-05
GO:0008094	MF	DNA-dependent ATPase activity	8.5974E-05
GO:0140097	MF	catalytic activity, acting on DNA	9.3587E-05
GO:0061629	MF	RNA polymerase II-specific DNA-binding transcription factor binding	0.00018296
GO:0003714	MF	transcription corepressor activity	0.00028647
GO:0042054	MF	histone methyltransferase activity	0.00028647
GO:0140030	MF	modification-dependent protein binding	0.00036025
GO:0016922	MF	nuclear receptor binding	0.00051054
GO:0035257	MF	nuclear hormone receptor binding	0.00051054
GO:0030695	MF	GTPase regulator activity	0.00051054
GO:0005096	MF	GTPase activator activity	0.00055999
GO:0042800	MF	histone methyltransferase activity (H3-K4 specific)	0.00055999
GO:0051427	MF	hormone receptor binding	0.00182932
GO:0003678	MF	DNA helicase activity	0.00268319
GO:0060589	MF	nucleoside-triphosphatase regulator activity	0.0034484
GO:0035258	MF	steroid hormone receptor binding	0.00346983

GO:0018024	MF	histone-lysine N-methyltransferase activity	0.00352063
GO:0031491	MF	nucleosome binding	0.0041934
GO:0017016	MF	Ras GTPase binding	0.00440051
GO:0019787	MF	ubiquitin-like protein transferase activity	0.00463441
GO:0008170	MF	N-methyltransferase activity	0.00463441
GO:0042826	MF	histone deacetylase binding	0.00463441
GO:0015631	MF	tubulin binding	0.005396
GO:0070491	MF	repressing transcription factor binding	0.005396
GO:0031267	MF	small GTPase binding	0.00606026
GO:0008276	MF	protein methyltransferase activity	0.00683418
GO:0017056	MF	structural constituent of nuclear pore	0.00683418
GO:0008022	MF	protein C-terminus binding	0.00711583
GO:0043138	MF	3'-5' DNA helicase activity	0.00711583
GO:0035197	MF	siRNA binding	0.00711583
GO:0008017	MF	microtubule binding	0.00833975
GO:0008536	MF	Ran GTPase binding	0.00850279
GO:0004843	MF	thiol-dependent ubiquitin-specific protease activity	0.01177545
GO:0101005	MF	ubiquitinyl hydrolase activity	0.01177545
GO:0008013	MF	beta-catenin binding	0.01225854
GO:0004674	MF	protein serine/threonine kinase activity	0.01257
GO:0031490	MF	chromatin DNA binding	0.01842329
GO:0016279	MF	protein-lysine N-methyltransferase activity	0.01842329
GO:0016278	MF	lysine N-methyltransferase activity	0.02006417
GO:0003777	MF	microtubule motor activity	0.02066509
GO:0005085	MF	guanyl-nucleotide exchange factor activity	0.02216274
GO:0016251	MF	RNA polymerase II general transcription initiation factor activity	0.02216274
GO:0004842	MF	ubiquitin-protein transferase activity	0.02216274
GO:0008242	MF	omega peptidase activity	0.023919
GO:0008168	MF	methyltransferase activity	0.023919
GO:0033613	MF	activating transcription factor binding	0.02399348
GO:0042974	MF	retinoic acid receptor binding	0.02678321
GO:0003697	MF	single-stranded DNA binding	0.0270122
GO:0070628	MF	proteasome binding	0.02863031
GO:0030374	MF	nuclear receptor transcription coactivator activity	0.03084688
GO:0008301	MF	DNA binding, bending	0.03397152
GO:0017116	MF	single-stranded DNA helicase activity	0.03397152
GO:0016741	MF	transferase activity, transferring one-carbon groups	0.03459773
GO:0030331	MF	estrogen receptor binding	0.0348867
GO:0140223	MF	general transcription initiation factor activity	0.03872058
GO:0019789	MF	SUMO transferase activity	0.0391844
GO:0070016	MF	armadillo repeat domain binding	0.04303244
GO:0008757	MF	S-adenosylmethionine-dependent methyltransferase activity	0.0439236
GO:0061980	MF	regulatory RNA binding	0.04579216
GO:0017048	MF	Rho GTPase binding	0.04729249

BP: Biological Process; CC: Cellular Component; MF: Molecular Function

Supplementary Table 5. Animal methods and Animal Research Reporting of In Vivo Experiments (ARRIVE) compliance.

Ethical statement	The in vivo study was conducted in agreement with the national and international guidelines and policies (European Economic Community Council Directive 86/609, OJ L 358, 1, December 12, 1987; Italian Legislative Decree 4.03.2014, n.26, *Gazzetta Ufficiale della Repubblica Italiana* no. 61, March 4, 2014) and were approved by the Italian Ministry of Health (N.270/2018-PR; N.1551/2020-PR). Mice were humanely sacrificed by CO_2 inhalation.
Study design	a. Intratibial injections in the left tibia of 10^5/0.01 ml PBS of MDA-MB231 cells MACS-sorted for HIGH or LOW expression on CXCR4, CD34 or TIE2:<br>b. Experimental groups: 5-week-old, female CD1 nu/nu mice<br>i. CXCR4^{LOW} (7 mice); CXCR4^{HIGH} (7 mice)<br>ii. CD34^{LOW} (7 mice); CD34^{HIGH} (7 mice)<br>iii. TIE2^{LOW} (7 mice); TIE2^{HIGH} (7 mice)<br>c. Experimental units:<br>i. Randomized groups of animals.<br>d. Samples that were not analyzed were stored and will be used for other projects, according to the 3Rs principles (reduce).
Experimental procedures	a. Bone metastasis generation: Mice were injected intratibially once with 10^5/0.01 ml PBS MDA-MB231 indicated in the Study design section. Treatments were done in the morning and ended before noon. Animals were monitored for a further 3 hours.<br>b. Anesthesia: Ketamine/xylazine cocktail (87.5 mg/kg ketamine, 12.5 mg/kg xylazine) ophthalmic ointment was applied to both eyes to prevent desiccation. To recover from anesthesia mice were placed in warm, clean, dry, quiet environment away from other animals. Commercially available surgical heating pads were used to warm up animals. Bedding material was replaced with toweling material to prevent bedding sticking to eyes or be inhaled while animals were recovering from anesthesia.<br>c. Euthanasia: At the end of the experiments, mice were euthanized by CO_2 inhalation.
Experimental animals	Mice used for in vivo experiments were CD1 nu/nu, females, age 5 weeks, weight 15-30 ± 3-5 gr.<br>Genotype: CD1-*Foxn1^{nu}*.<br>Animals lack the thymus, are unable to produce T-cells, and are therefore immunodeficient.
Housing and husbandry	Animal facility: standard.<br>Temperature 20-24°C.<br>Diet: access to food and water *ad libitum*, normal diet (Mucedola code: 3KE25).<br>Dark/light cycle: 12/12 hours.<br>Humidity: 60 ± 5%<br>Cage: plastic.<br>Cage companions, 3 adults/cage, genders were not mixed.<br>Bedding material: high adsorbing power, without dust, changed every week.<br>Environmental enrichment was done with sterile material.
Sample size	Seven-7 animals/group were used for the experiments.<br>The sample size was calculated for all the experiments using dedicated software (SigmaPlot v12), based on the expected differences.<br>The efficacy of the methods used, and our long-standing experience allowed us to obtain significant results with relatively little sample sizes, according to the 3Rs principles.
Allocating animals to	Animals were assigned to groups after randomization.

experimental groups	
Experimental outcomes	To investigate the importance of the CXCR4, CD34 or TIE2 in tumour dormancy.
Statistical	Statistical analysis was performed by the Student's *t*-test and by the Benjamini-Hochberg adjustment p value procedure. The statistical methods are indicated in the figure and table legends. The p-values are indicated in the figures. A p value <0.05 was conventionally considered statistically significant.

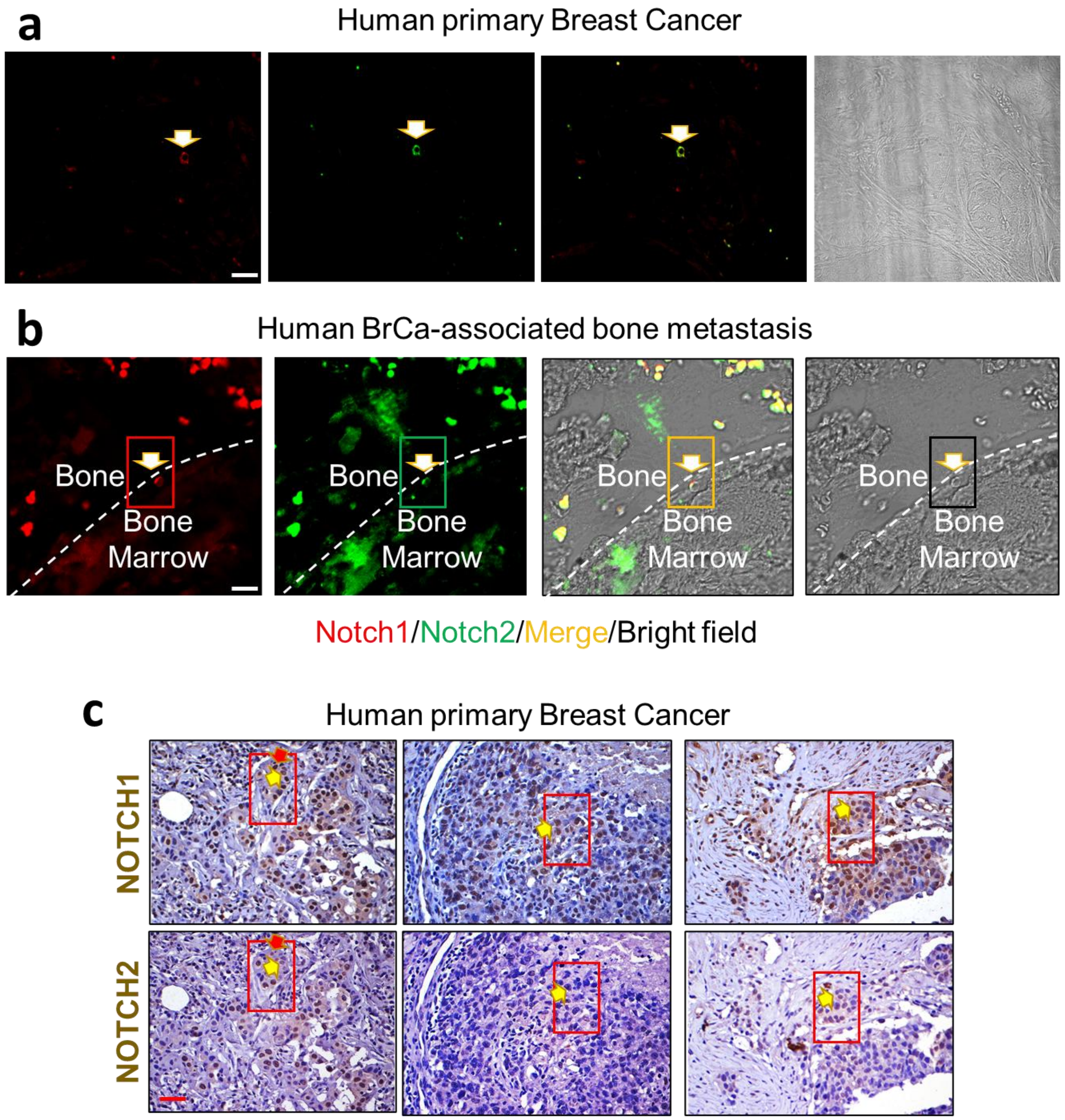

Supplementary Figure 1. Low magnification of imagines depicted in main Figure 1 (a), (b) and (c). Arrows: cells of interest. Rectangles: areas shown in figure 1 panels. Bar=30µm.

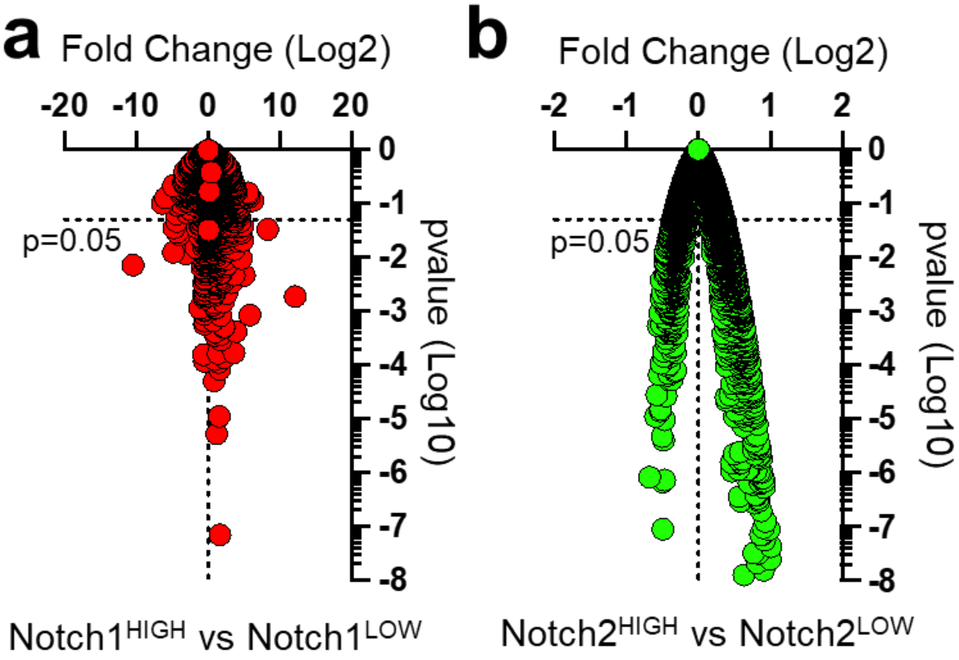

Supplementary Figure 2. Differentially expressed genes in MDA-MB231 cells. RNAdSeq showing differentially expressed genes in MACS-sorted **(a)** Notch1^{HIGH} vs Notch1^{LOW} and **(b)** in Notch2^{HIGH} vs Notch2^{LOW} MDA-MB231 cells. Results are the mean$\pm$SD of 3 independent experiments evaluated by the Benjamini-Hochberg adjustment p value procedure.

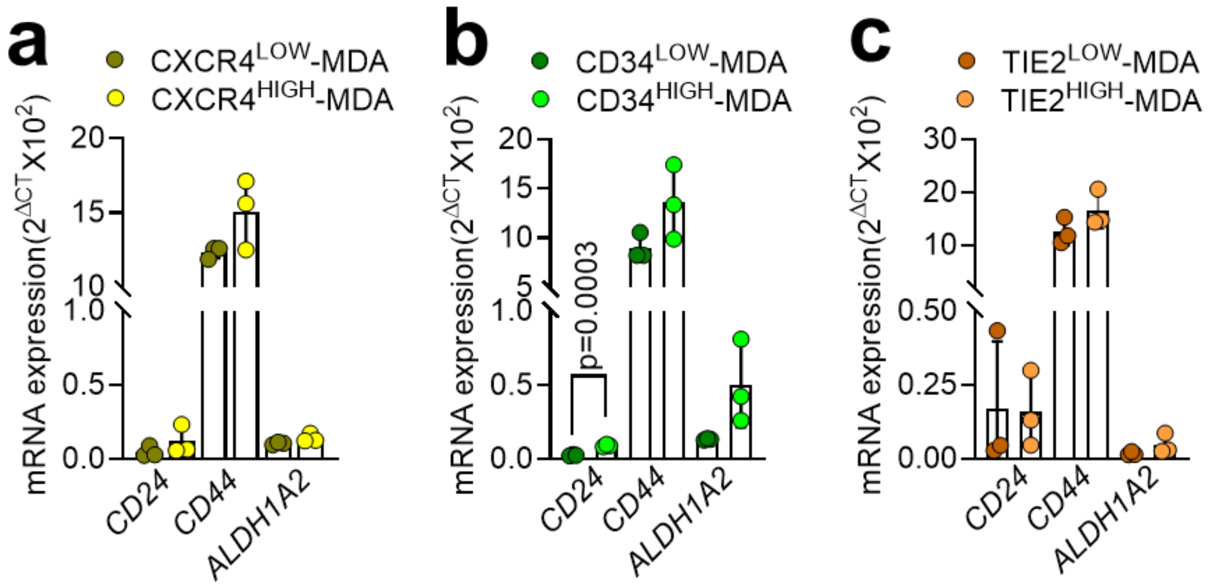

Supplementary Figure 3. Expression of cancer cell genes. Transcriptional expression of the cancer cell genes CD24, CD44 and ALDH1A2 genes in **(a)** CXCR4^{LOW} and CXCR4^{HIGH}, **(b)** CD34^{LOW} and CDR4^{HIGH} and **(c)** TIE2^{LOW} and TIE2^{HIGH} MDA-MB231 cells. Results are the mean$\pm$SD of 3 independent experiments. Statistics: unpaired t test.

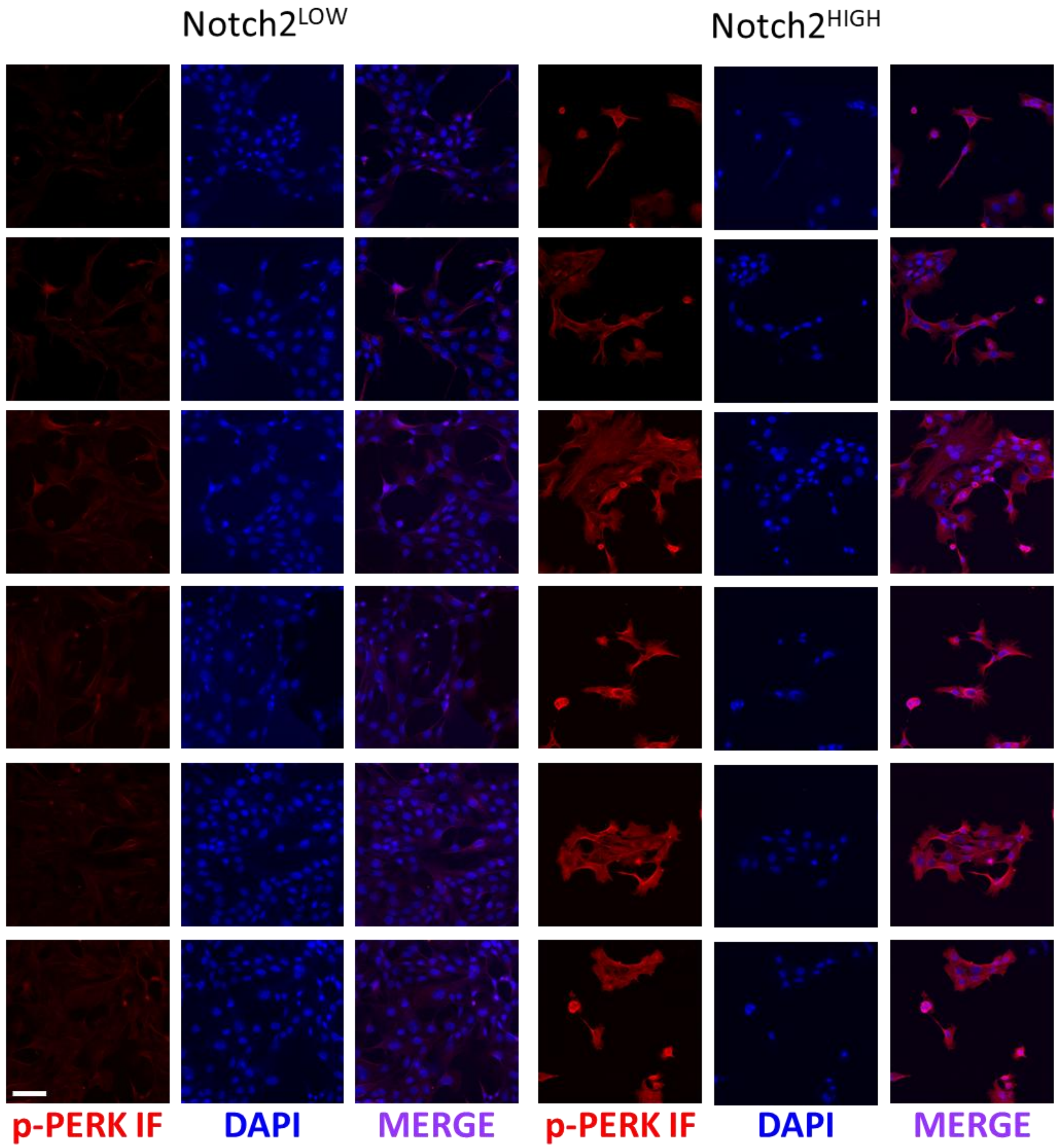

Supplementary Figure 4. p-PERK immunofluorescence staining. Representative image panel illustrating p-PERK expression detected by immunofluorescence in Nocth2^{LOW} and Notch2^{HIGH} cells along with nuclear DAPI staining. (Bar=15 µm).

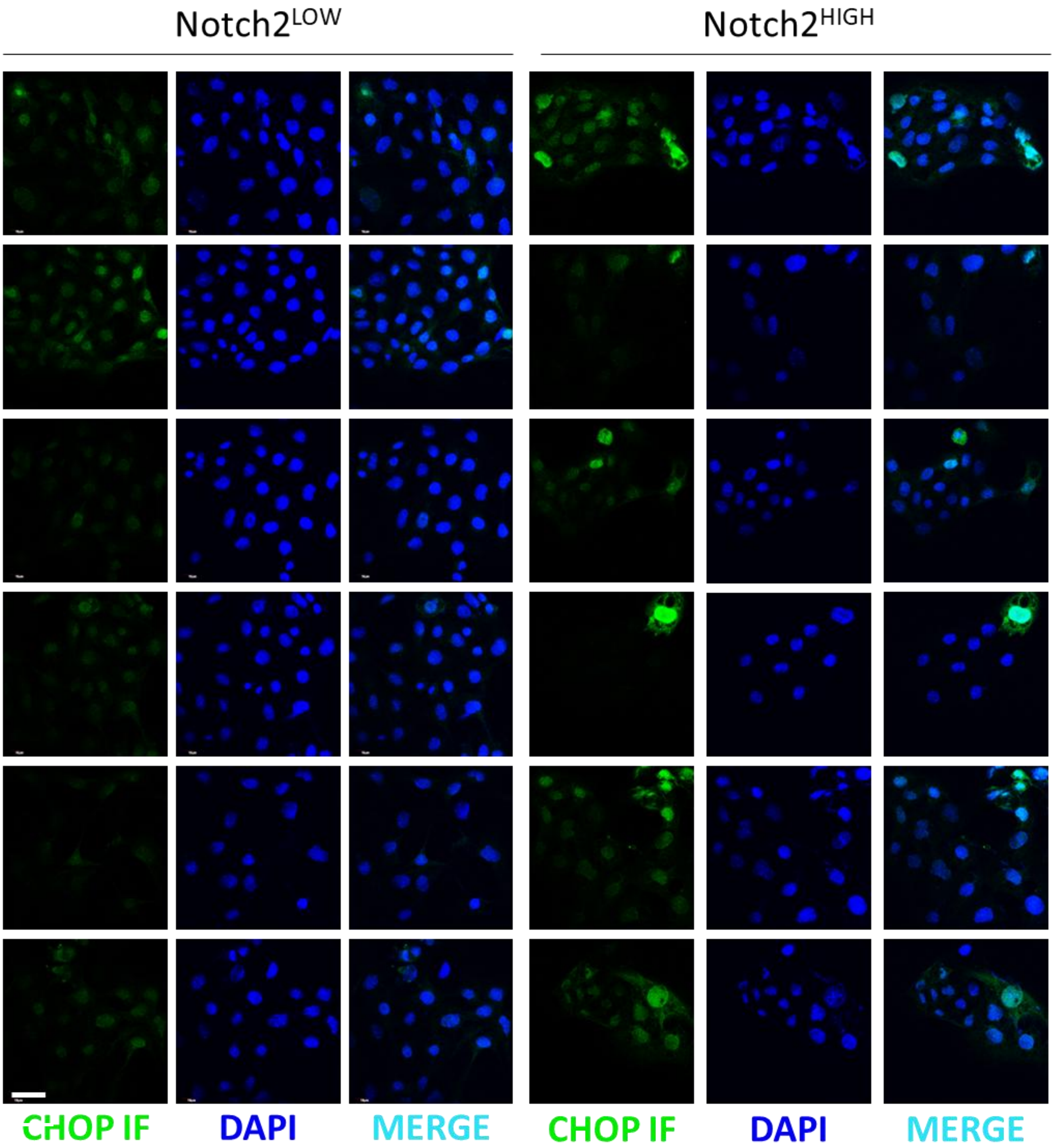

Supplementary Figure 5. CHOP immunofluorescence staining. Representative image panel illustrating CHOP expression detected by immunofluorescence in Nocth2^{LOW} and Notch2^{HIGH} cells along with nuclear DAPI staining. (Bar=15 µm).

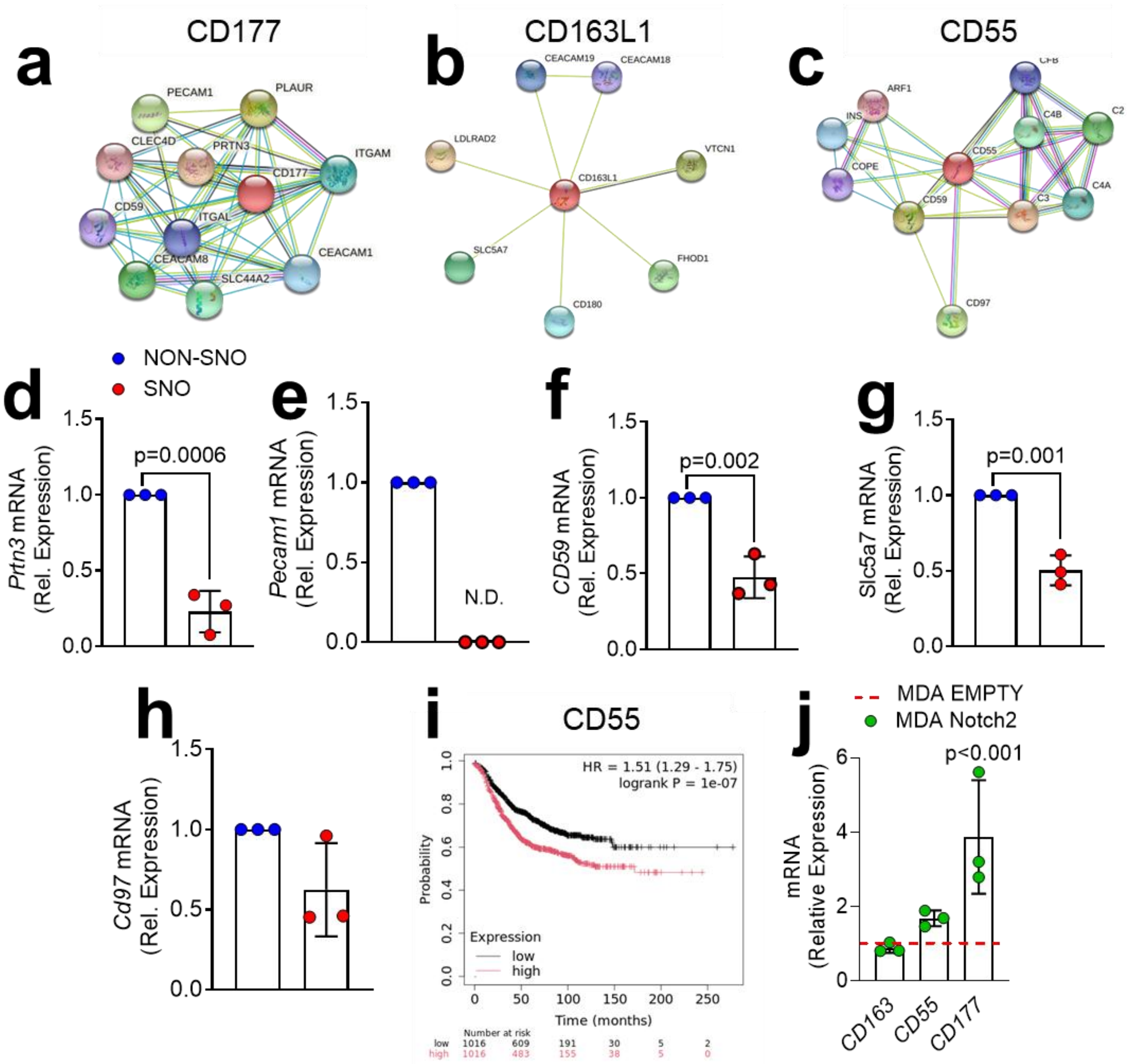

Supplementary Figure 6. Expression of CD177, CD163L1 and CD55 interactants in NON-SNO and SNO cells. Molecular network extrapolated from the RNAdSeq data illustrating **(a)** CD177, **(b)** CD163L1 and **(c)** CD55 interactants expressed by NON-SNO and SNO cells. **(d)** Transcriptional expression of *Prtn3*, **(e)** *Pecam1*, **(f)** *CD59*, **(g)** *Slc5a7* and **(h)** *CD97* in NON-SNO and SNO cells. **(i)** Kaplan-Meier Plot of a cohort of BrCa patients expressing low and high levels of CD55. **(j)** Transcriptional expression of *CD163*, *CD55* and *CD177* in MDA-MB231 cells transfected with an empty vector or with Notch2 vector. Results are the mean±SD of 3 independent experiments. Statistics: unpaired t test.

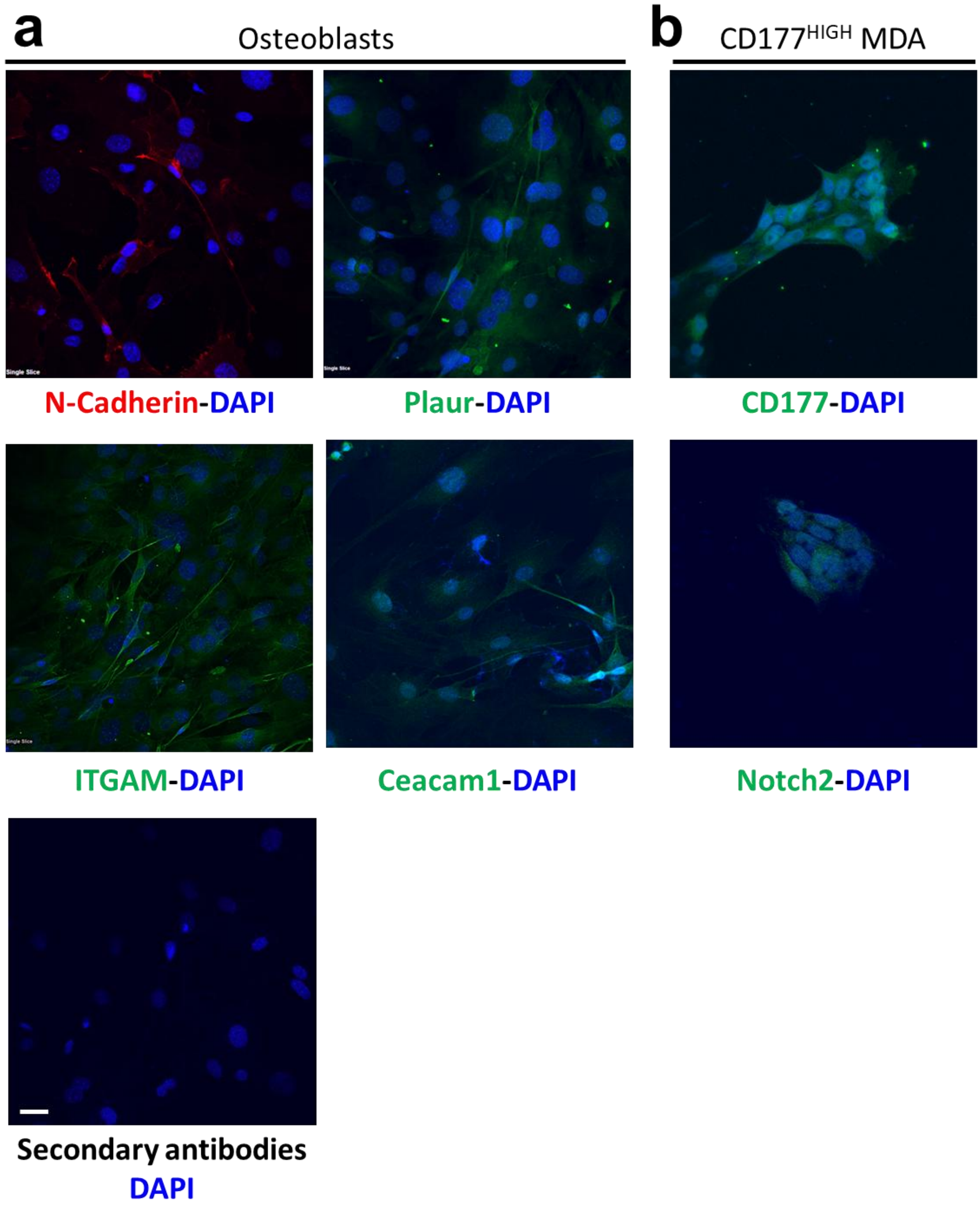

Supplementary Figure 7. Immunofluorescence staining of osteoblasts and MDA-MB231 BrCa cells. **(a)** Osteoblasts and **(b)** CD177^{HIGH} MDA-MB231cells were cultured in standard conditions and immunostained for the indicated proteins. Bar=10µm.
